# Emergent Properties of Microbial Activity in Heterogeneous Soil Microenvironments: Different Research Approaches Are Slowly Converging, Yet Major Challenges Remain

**DOI:** 10.3389/fmicb.2018.01929

**Published:** 2018-08-27

**Authors:** Philippe C. Baveye, Wilfred Otten, Alexandra Kravchenko, María Balseiro-Romero, Éléonore Beckers, Maha Chalhoub, Christophe Darnault, Thilo Eickhorst, Patricia Garnier, Simona Hapca, Serkan Kiranyaz, Olivier Monga, Carsten W. Mueller, Naoise Nunan, Valérie Pot, Steffen Schlüter, Hannes Schmidt, Hans-Jörg Vogel

**Affiliations:** ^1^UMR ECOSYS, AgroParisTech, Université Paris-Saclay, Thiverval-Grignon, rance; ^2^School of Water, Energy and Environment, Cranfield University, Cranfield, United Kingdom; ^3^Department of Plant, Soil and Microbial Sciences, Michigan State University, East Lansing, MI, United States; ^4^Department of Soil Science and Agricultural Chemistry, Centre for Research in Environmental Technologies, Universidade de Santiago de Compostela, Santiago de Compostela, Spain; ^5^Soil–Water–Plant Exchanges, Terra Research Centre, BIOSE, Gembloux Agro-Bio Tech, University of Liège, Gembloux, Belgium; ^6^UMR ECOSYS, INRA, Université Paris-Saclay, Thiverval-Grignon, France; ^7^Laboratory of Hydrogeoscience and Biological Engineering, L.G. Rich Environmental Laboratory, Department of Environmental Engineering and Earth Sciences, Clemson University, Clemson, SC, United States; ^8^Faculty 2 Biology/Chemistry, University of Bremen, Bremen, Germany; ^9^Dundee Epidemiology and Biostatistics Unit, School of Medicine, University of Dundee, Dundee, United Kingdom; ^10^Department of Electrical Engineering, Qatar University, Doha, Qatar; ^11^Institut de Recherche pour le Développement, Bondy, France; ^12^Lehrstuhl für Bodenkunde, Technical University of Munich, Freising, Germany; ^13^Institute of Ecology and Environmental Sciences – Paris, Sorbonne Universités, CNRS, IRD, INRA, P7, UPEC, Paris, France; ^14^Soil System Science, Helmholtz-Zentrum für Umweltforschung GmbH – UFZ, Leipzig, Germany; ^15^Terrestrial Ecosystem Research, Department of Microbiology and Ecosystem Science, Research Network ‘Chemistry meets Microbiology’, University of Vienna, Vienna, Austria; ^16^Institute of Soil Science and Plant Nutrition, Martin Luther University of Halle-Wittenberg, Halle, Germany

**Keywords:** soil microbiology, biodiversity, upscaling, tomography, X-ray computed, NanoSIMS imaging, single-cell genomics

## Abstract

Over the last 60 years, soil microbiologists have accumulated a wealth of experimental data showing that the bulk, macroscopic parameters (e.g., granulometry, pH, soil organic matter, and biomass contents) commonly used to characterize soils provide insufficient information to describe quantitatively the activity of soil microorganisms and some of its outcomes, like the emission of greenhouse gasses. Clearly, new, more appropriate macroscopic parameters are needed, which reflect better the spatial heterogeneity of soils at the microscale (i.e., the pore scale) that is commensurate with the habitat of many microorganisms. For a long time, spectroscopic and microscopic tools were lacking to quantify processes at that scale, but major technological advances over the last 15 years have made suitable equipment available to researchers. In this context, the objective of the present article is to review progress achieved to date in the significant research program that has ensued. This program can be rationalized as a sequence of steps, namely the quantification and modeling of the physical-, (bio)chemical-, and microbiological properties of soils, the integration of these different perspectives into a unified theory, its upscaling to the macroscopic scale, and, eventually, the development of new approaches to measure macroscopic soil characteristics. At this stage, significant progress has been achieved on the physical front, and to a lesser extent on the (bio)chemical one as well, both in terms of experiments and modeling. With regard to the microbial aspects, although a lot of work has been devoted to the modeling of bacterial and fungal activity in soils at the pore scale, the appropriateness of model assumptions cannot be readily assessed because of the scarcity of relevant experimental data. For significant progress to be made, it is crucial to make sure that research on the microbial components of soil systems does not keep lagging behind the work on the physical and (bio)chemical characteristics. Concerning the subsequent steps in the program, very little integration of the various disciplinary perspectives has occurred so far, and, as a result, researchers have not yet been able to tackle the scaling up to the macroscopic level. Many challenges, some of them daunting, remain on the path ahead. Fortunately, a number of these challenges may be resolved by brand new measuring equipment that will become commercially available in the very near future.

## Introduction

Over the last decade, soils have become increasingly central to a number of crucial debates on issues of great societal concern. Because they contain a huge amount of carbon, soils could lead to a dramatic acceleration of global climate change, as mean temperatures increase and rainfall patterns are altered (Baveye, 2007; Baveye et al., 2011; Hamdi et al., 2013; Crowther et al., 2016). The idea, advocated by some (Paustian et al., 1997; Lal and Bruce, 1999), that on the contrary, with proper management, soils could store even more carbon than at present, and thereby help mitigate the production of greenhouse gasses resulting from the consumption of fossil fuels, has been adopted enthusiastically by politicians in a number of countries but has stirred intense discussions among scientists (Powlson et al., 2011; Cheng et al., 2012; Dungait et al., 2012; Kowalchuk, 2012; Verbruggen et al., 2012; Minasny et al., 2017, 2018; van Groenigen et al., 2017; Baveye et al., 2018; White et al., 2018). At the same time, humanity is faced with the prospect of having to significantly increase food production to feed the world population, which is expected to rise to 9 or 10 billion people by 2050 (Godfray et al., 2010). Since soil and water resources are already used at the maximum level of what some consider ecologically safe, a consensus seems to be emerging that as long as the focus is kept on land-based agricultural production, the best option to insure food security lies in exploiting plant-microbe partnerships to improve biomass production (Weyens et al., 2009; Glick, 2012, 2014; Blaser et al., 2016), or in stimulating so-called plant-soil feedback processes, whereby plants induce soil microbial communities to release nutrients and store water in the rhizosphere (Sposito, 2013; Baveye, 2015). In addition, even though the issue of soil contamination does not appear at the moment to be at the forefront of environmental concerns in many countries, the question remains of what to do with millions of severely polluted sites around the globe, especially given the fact that this number is ever increasing, as a result of practices like shale gas production (Baveye, 2013c; Meckenstock et al., 2015). Given the prospect of a progressive warming of soils in decades to come, renewed threats caused by soil contamination will undoubtedly need to be addressed at some point in the near future.

The intimately connected microbial and physico-chemical processes at the core of all these soil-related issues have posed daunting challenges to researchers. Until a decade ago, in spite of sustained research efforts, progress was very slow or even non-existent, and in several cases serious hurdles arose, which no one had anticipated. Kirschbaum (2006) admitted that in the 10 years prior to the publication of his review of the field, no real advance had been made in understanding and predicting quantitatively the effect of temperature on the decomposition of soil organic matter (OM). Available models also routinely underestimated the pulses of CO_2_ flux occurring when large rainfall events follow drafts (Blagodatsky and Smith, 2012; Evans et al., 2016). Recent work by Rabot et al. (2015) suggests that many of the previous measurements of the production by soil bacteria and fungi of nitrous oxide, a very potent greenhouse gas (Laughlin and Stevens, 2002; Crenshaw et al., 2008; Hu et al., 2015), probably missed very short emission bursts that occur at the onset of drying of soils, and therefore underestimated total N_2_O production by soils. Concomitantly, research on carbon sequestration in soils provided evidence of the problematic “priming” effect, identified early on (Macura et al., 1965; Arsjad and Giddens, 1966), but routinely overlooked until a decade ago (Fontaine et al., 2007; Kuzyakov, 2010; Tian et al., 2015) and still poorly understood (Nunan et al., 2015; van der Wal and de Boer, 2017). Through this effect, the addition of fresh OM to soils can lead to the mineralization of very old humic substances, previously thought to be utterly stable and recalcitrant to further degradation. In a similar fashion, in polluted soils, experiments showed that a slight change, for example brought about by the addition of a source of nutrients for microorganisms, could easily make supposedly “sequestered” contaminants once again bioavailable (Li et al., 2005). Some of these areas of ignorance remain “terra incognita” at this point, even with regard to the much ballyhooed biodiversity of soils (Baveye et al., 2016a,b). There is still no satisfactory explanation for the observation, made more than 60 years ago, that the mineralization of soil OM continues at the same rate even if 90% of soil microorganisms are wiped out by CHCl_3_ fumigation (Jenkinson, 1966; Powlson et al., 2017; Baveye, 2018). A final example of a situation where our understanding of soil systems is still insufficient is related to the links between the diversity of soil microbial communities and various soil parameters. Some authors have found a close correlation between this diversity and specific parameters, like soil pH (Fierer and Jackson, 2006), but more detailed statistical analyses sometimes present a different picture. In a recent study, Terrat et al. (2017) use some of the most sophisticated molecular techniques currently available to analyze the biodiversity of soil samples across France, and try to relate it to various parameters of soils and of their environment. The results are systematically underwhelming. They find that less than half (48.2%) of the observed variance of the biodiversity could be accounted for by using soil parameters that are routinely measured. Clearly, at least in this particular study, something fundamental about soils is being missed.

In virtually all these instances, a common observation is that soil samples that appear alike in most of their overall measured characteristics can behave very differently, making replicated observations and good correlations difficult to achieve. Obviously, it is not sufficient to describe soils solely on the basis of traditional macroscopic measurements, such as the volumetric water content, microbial density, or contaminant concentration. Quantitative information on the spatial heterogeneity manifested at the micron scale, at which microorganisms operate, is also absolutely required.

In some respects, this is not as novel a perspective as it may appear. In another era, in literature that unfortunately seems to have become largely ignored since, soil microbiologists already reached the same conclusion. Sixty years ago, Rovira and Greacen (1957) subjected moist soil samples to compression and shearing to simulate tillage, and concluded, after ruling out other possible explanations, that the enhanced oxygen consumption observed in the soils after disruption was due to exposure of organisms to OM that was previously inaccessible to them. These and a number of other early observations pointing in the same direction prompted Alexander (1964, p. 219) to conclude that “microorganisms apparently in the same habitat are, in fact, often exposed to entirely different environmental influences and population pressures. To understand the forces actually affecting the organisms, a microenvironmental concept rather than the gross macroscopic view of interactions must be adopted.” The review by Griffith (1965) of the extensive work carried out in the 40s and 50s on the opposite effect of microorganisms on their physical environment, and in particular on the development of soil architecture, also raises many questions that could be addressed only from a microscopic perspective. Experimental evidence obtained since the mid-sixties has provided steadily strengthening support for this perspective (Hattori, 1973; Cheshire, 1977; Elliott et al., 1980; Tiedje et al., 1984; Stotzky, 1986; Crozat et al., 1987; Darrah et al., 1987; Parkin et al., 1987; Postma and Altemuller, 1990; Postma and van Veen, 1990; Killham et al., 1993; Renault and Stengel, 1993; Strong et al., 1997; Wachinger et al., 2000; Chenu and Stotzky, 2001; Attard et al., 2011; Chapman et al., 2012; Johnson et al., 2013; Vos et al., 2013; Uroz et al., 2015; Xun et al., 2015; Barcenas-Moreno et al., 2016; Keiluweit et al., 2017, 2018).

In the 50s and 60s, very little could be done to come up with better measurements, unfortunately. Alexander (1964, p. 219), again, observed that “because of inherent technical difficulties in biochemical experimentation at the microscopic level, progress in understanding of the microenvironment has been painfully slow.” Even though more and more experiments over the years confirmed the significance of microenvironments, for a long time it was not feasible practically to characterize them in quantitative terms. The advent of transmission or scanning electron microscopes, and later of confocal laser microscopes as well, provided a wealth of qualitative information about microbial habitats in the form of micrographs of increasingly high quality (Foster, 1988; Vandevivere and Baveye, 1992a,b,c,d; DeLeo et al., 1997; Baveye et al., 1998), but the lack of related quantitative data prevented for several decades the development of satisfactory predictive models of soil microbial processes, accounting explicitly for the microheterogeneity of soils.

This situation has changed dramatically in the last decade and is continuing to evolve at a rapid pace. Significant technological advances have provided soil researchers, for example, with routine access to X-ray computed tomography (CT) systems, which provide increasingly reliable information about the geometry of pores and solids in soils at resolutions as small as 0.05 μm. Progress in near-edge X-ray spectromicroscopy (NEXAFS), scanning transmission X-ray microscopy (STXM), X-ray absorption spectroscopy, micro-fluorescence spectroscopy, and Nano-SIMS, applied to soil thin sections, has led to observations of sharp spatial heterogeneity in the chemical make-up of soils over minute distances, and in the accumulation of trace metals. Significant advances related to biological markers now allow specific bacteria to be identified in soils, and their spatial distribution at the micrometer scale to be determined in thin sections. This information can be translated into 3-dimensional distributions using recently developed statistical algorithms. In addition, very efficient modeling tools, like the Lattice-Boltzmann approach, allow the description of transport and physico-chemical processes occurring in soil pores at scales that are directly relevant to microorganisms, whereas individual-based or agent-based models, also developing rapidly, can describe the dynamics of microorganisms inhabiting the pore space (Gras et al., 2010, 2011; Muci et al., 2012; Hellweger et al., 2016; Kim and Or, 2016).

In the last few years, the application of each of these technologies and modeling methods to soils has been the object of a sizeable literature. Progress achieved in the use of each technology has already been expertly reviewed (O’Donnell et al., 2007; Taina et al., 2008; Young et al., 2008; Behrens et al., 2012; Rennert et al., 2012; Helliwell et al., 2013; Tuller et al., 2013; Wildenschild and Sheppard, 2013; Schlüter et al., 2014; Calistru and Jitareanu, 2015; Kuzyakov and Blagodatskaya, 2015; Prosser, 2015; Roose et al., 2016; Xiong et al., 2016; Totsche et al., 2017). For some technologies, since advances are extremely rapid, it would be useful, conceivably, to provide an updated coverage of recent work, and no doubt new reviews will fill the gaps in the near future. Yet, a different type of critical overview might be even more fruitful at this stage, one that keeps sight firmly on what started out as the ultimate goal of the research: A thorough understanding of what one needs to measure at the macroscale in order to adequately describe emergent microbial processes. Instead of surveying the increasingly widespread application of specific technologies to soils, it is worth taking a step back and analyzing how the use of these technologies and their continual improvements help us, or are expected to help us, move steadily on paths leading to the goal we seek. For each path, we can try to assess how far along we are at present and, to the best of our knowledge, to estimate how much distance remains to be covered. Also, since at the scale of bacterial and archaeal cells, it is virtually impossible to dissociate physical, (bio)chemical, and biological aspects of soils, another key point of interest is the extent to which the combined uses of different technologies, meant to access information on these complementary aspects, make us now, or at least promise to make us soon, converge consistently toward meaningful insights. In this reflection on what remains to be done, it makes sense to try to gauge as well how much assistance we could derive from measurement technologies that are barely emerging at the moment but will in all likelihood become routinely available to us in the next few years. It is to scrutiny along these different directions that the present review article is devoted.

## Keeping One’s Eyes on the Ultimate Goal

First things first. As a famous microbiologist once wrote, “without the proper technological advances the road ahead is blocked. Without a proper vision, there is no road ahead” (Woese, 2004). So, it is vital to start from a clear perception of the goal that is being pursued, and then outline what paths lead to it. As pointed out above, it has been known for half a century at least that the type of macroscopic measurements that are carried out routinely on soils and sediments at the moment do not inform in a satisfactory way about the parameters that appear to be controlling the activity of microorganisms in these systems. Experience has shown clearly that knowledge of, e.g., the total microbial biomass and the total amount of OM present in a given volume of soil or sediment does not allow us to make reliable predictions about the activity of microorganisms or the fate of OM. Somehow, our usual measurements do not capture enough of the huge complexity that soils manifest at the microscopic scale to enable us to predict accurately various properties of soils, like the activity of microorganisms, at the macroscopic scale.

To describe the process by which microscale heterogeneity influences and generates macroscopic behaviors, researchers have used alternatively the terms of “emergence” (Holland, 1990; Addiscott, 2011) or “self-organization” (Smagin, 1989; Hallet, 1990; Phillips, 1995, 2000; Manson, 2001; Young and Crawford, 2004; Barot et al., 2007; Lavelle et al., 2007, 2016; Ebrahimi and Or, 2016; Tecon and Or, 2017a,b). For a number of reasons, explained in detail in **Appendix 1 (Supplementary Information)**, “emergence,” implying a reality that is *less* than the sum of its parts and is therefore much simpler to describe, is far more appropriate than the term of “self-organization” to describe the type of soil-borne processes on which this review article focuses. In the following, we shall therefore refer consistently to “emergence.”

**FIGURE 1:**
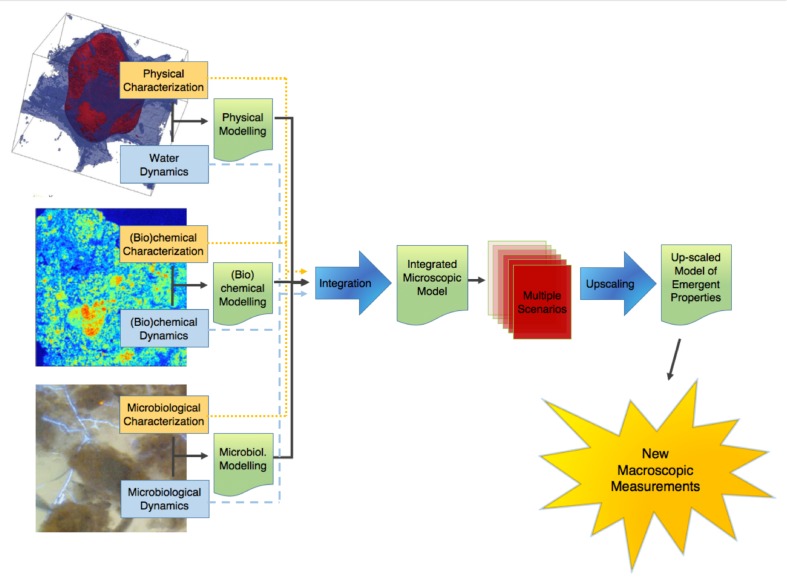
Schematic representation of the sequence of steps in the research on the emergent properties of soils, leading from a characterization of the various properties and dynamics at the microscale, onward to an upscaled macroscopic model, and finally to the ultimate goal of identifying macroscopic measurements that can be carried out routinely.

This point of terminology being resolved, the crux of the matter is that information of an entirely different nature than that currently available is needed to describe soil microbial processes adequately. We clearly need new macroscopic measurements. There are probably different ways to envisage the paths that will lead us eventually to this “Holy Grail.” **Figure 1** proposes one of these perspectives, which has served as a general strategy map to a number of us in our research efforts. It starts on the left with information about basic soil features. What we understand at this point of emergent processes in soils indicates that this topic has (at least) three clear, resolutely interdependent facets, associated, respectively, with physical-, (bio)chemical-, and microbiological aspects of soils. For each of them, it is crucial to gather experimental information, either on static properties (dealt with in the boxes “physical characterization,” etc.), or on their dynamics. Alongside this evidence gathering, it is also important to develop theoretical and modeling frameworks that encapsulate experimental information and allow predictions to be made. In each case, experimental data should serve to refine theories and models, which in turn (e.g., through sensitivity analyses) can provide guidance in the procurement of additional data. The outcome of this type of iterative approach, hopefully, is a satisfactory description of each dynamic, which can then be integrated at first pairwise, and eventually all together, into a comprehensive model of soil processes at the microscale.

At that point, we are still somewhat far from the goal. Indeed, when this integrated model becomes available, running it on any given soil sample will require a tremendous amount of microscale information, which may take weeks or even months to gather, not to mention that the integrated model itself will likely take quite some time to run, which means that with this integrated microscale description of soils, only very few soil samples will ever be characterized and modeled. What we need instead is to come up with simple *macroscopic* measurements that can be carried out *routinely*.

One way to find out what these macroscopic measurements should be is suggested in **Figure 1**. It consists of expanding the available experimental database by simulating many different scenarios under different conditions of microscale heterogeneity of the soils, and of their properties. From these multiple scenarios, one can try to find out how one can simplify the description, in other words upscale the microscale model to the macroscale, while making sure that, in the process, the macroscopic parameters that appear in the resulting upscaled description are amenable to routine measurement in practice. This prospect of course rests on the assumption, at this stage very much open, that the simplification implied by the notion of “emergence” indeed occurs in soils. As discussed later, there is fortunately some circumstantial evidence that such simplification can be expected.

The research program, depicted in **Figure 1**, comprises a number of clear paths, which are discussed in the following. To the extent that some of the steps along these paths involve advanced technologies and elaborate methods of analysis or simulation, there is a definite risk of drift, i.e., to focus excessively on tools, perfect them, and progressively forget over time the reason for doing all this work in the first place, as one could argue has unfortunately happened occasionally in the past in other contexts in soil science (Baveye and Laba, 2015). One might argue that switching progressively from actual soils to very coarse sands or collections of clean 500 μm (or bigger) glass beads constitutes an example of such a drift. These systems admittedly pose far fewer technological challenges, which enable researchers to identify and isolate various microscale mechanisms, but, as experience acquired in the past (e.g., in the sixties, when glass beads were used to research several soil processes, like water retention hysteresis) has shown, the relevance of the information acquired in these idealized systems for the functioning of real soils is unclear, at best. To avoid such drift, as much as possible, the descriptions of the various paths of **Figure 1** will focus exclusively on progress made to date with actual, living *soils*, in all their wonderful complexity and messiness.

One last comment that needs to be made before we embark in the description of the program of **Figure 1** is that there is no reason to be so wedded to it as not to be open to alternatives that may surface. If tomorrow, an experimentalist comes up with a robust empirical relationship among novel macroscopic measurements, similar to what is envisaged as the ultimate outcome of the program of **Figure 1**, every researcher interested in the field should probably rejoice, change gear, and adopt an entirely different perspective, for example to try to understand why the solution works. This is reminiscent of the debate about top-down vs. bottom-up approaches in hydrology (Basu et al., 2011; Baveye and Laba, 2015). Regardless of how strongly held one’s philosophical beliefs are, what matters most is to find a satisfactory answer to a number of questions, not necessarily the manner in which the answers are obtained. This being said, no experimentalist has stepped forward yet with a ready answer, and the stepwise plan of **Figure 1** appears to be our best bet at this point to ever obtain one in the limited time we have to do so.

## Progress on the Physical Front

### Computed Tomography and Image Processing

Any overview of the quantitative research of the past 10 years on microscale processes in soils needs to start with their physical characterization. Indeed, soil physicists have undeniably led the charge. The pioneering work carried out in the early 1980s with medical and custom-made X-ray and gamma-ray computed tomography systems brought to the attention of the soil physics community the potential of this technology, then still in its infancy (Petrovic et al., 1982; Hainsworth and Aylmore, 1983; Crestana et al., 1985; Pires et al., 2010). The low (millimeter) resolution of scanners available at the time enabled researchers to characterize the geometry of macropores (e.g., earthworm burrows) in soils (Warner et al., 1989; Joschko et al., 1991; Heijs et al., 1995; Capowiez et al., 1998; Rogasik et al., 2003; Luo et al., 2010), but was much too coarse to provide information relevant to microorganisms. In the mid-1990s, various synchrotron facilities around the world began to devote beam time to soils, and researchers immediately took advantage of the significantly higher spatial resolution (down to a few μm) these facilities afforded, as well as the fact that the synchrotron X-ray beams are monochromatic (single-energy) (Anderson and Hopmans, 1994; Spanne et al., 1994; Garnier et al., 1998; Wildenschild et al., 2002; Feeney et al., 2006). However, access to synchrotron beam time was, and still is to a large extent, somewhat scarce and difficult to obtain, so that the extent of adoption of synchrotron-X-ray tomography has remained limited. The commercialization, around 2002, of the first tabletop, non-medical X-ray tomography systems, which were not excessively onerous and could therefore be entirely dedicated to soil science research, marked the beginning of a new era. The X-rays produced by these machines are polychromatic (i.e., are a mixture of X-rays of different energies), which in a number of ways is a disadvantage compared to the monochromatic X-ray produced by synchrotrons, but the resolution of these tabletop scanners has steadily improved since 2002 and several machines now allow resolutions that, in small soil samples of a few cm^3^, can be as low as 0.3 μm, i.e., commensurate with the resolution afforded by synchrotrons (Voltolini et al., 2017) and with the size of some of the “ultra-small” bacteria and archaea found in soils. The very high resolution of X-ray CT has for a time at least made other types of measuring instruments, like dual-energy gamma-ray scanners, neutron radiography, or nuclear magnetic resonance micro-imaging systems, fall off the radar screen, at least in applications to soils. Nevertheless, as we shall see later, these instruments afford advantages over X-ray CT, and are therefore likely to play a more significant role in the future.

The “3-dimensional” soil images that CT scanners provide are in fact stacks of 2-dimensional, grayscale images associated with virtual slices within the soil sample. Very early on in the use of these images, researchers came to the conclusion that these grayscale images would not be very useful to quantify the geometry of the soil pore space and that it was necessary to derive binary (black and white) images from the original grayscale ones, a process alternatively referred to as “thresholding” or “segmentation.” Significant progress has occurred over the years in how this thresholding is approached. Initially, it was carried out slice by slice, either manually by simple visual inspection (“eye-balling”) or with the assistance of one of a number of available 2D algorithms (e.g., Nunan et al., 2006). The first improvement consisted of thresholding the whole 3-d image at once, using an algorithm to calculate a unique, global threshold value. Then, various researchers showed that in the presence of textural heterogeneities (e.g., stones) within the samples, it was preferable to instead use local thresholds, which can vary from location to location within a sample (Iassonov et al., 2009; Schlüter et al., 2014). Up to that point, all thresholding algorithms required operator input, to adjust one or more parameters. This introduced unavoidable subjectivity in the process, which in principle would make it improbable for different individuals to threshold a given soil sample the same way, or even for a single individual to threshold different soil samples (e.g., associated with different agricultural practices or with successive times) in a consistent manner (Baveye et al., 2010).

The question of objectivity in the generation of X-ray CT images of soils is in fact much broader than just this issue regarding thresholding/segmentation. Indeed, as a number of authors have pointed out (Vaz et al., 2011; Houston et al., 2013b), the process of obtaining CT images of soils requires many decisions to be made by operators, concerning in particular the value of scanning parameters (e.g., energy level, choice of filter, scanning resolution), the selection of one among a number of alternative image reconstruction and artifact correction algorithms, the format (8- or 16 bit) used to store the images, and the use of a method to increase image sharpness or reduce the noise that is unavoidably present in the images after reconstruction. As with thresholding 10 years ago, different groups, and sometimes even different individuals within a group, adopt alternative perspectives with respect to the various decisions that need to be made by operators, which can lead to sometimes significant differences in some of the metrics that are associated eventually with CT images (see, e.g., Houston et al., 2013b). Nevertheless, at this point, there appears to be no effort underway to develop a set of materials that could be used as “scanning standards,” as suggested by Baveye et al. (2010), or simply to standardize analyzes. One way out of the difficulty would be to document exhaustively the parameter values used at each and every step of the image acquisition process, as well as, through detailed sensitivity analyses, the extent to which conclusions that are reached on the basis of CT images are affected by these parameter values.

Nevertheless, recognition a few years ago that the subjectivity in thresholding operations and in the manipulation of CT images could be substantial, prompted the development of a number of automated thresholding algorithms requiring no operator input (Schlüter et al., 2010; Hapca et al., 2013; Houston et al., 2013a), regardless of the level of “supervision” (learning from training data) adopted. These objective algorithms have been used in a number of investigations (e.g., Beckers et al., 2014a,b; Houston et al., 2017), and new algorithms are appearing that do not require any parameter tuning (e.g., West et al., 2018), but so far they have not stopped the development of operator-dependent approaches (Kulkarni et al., 2012; Hashemi et al., 2014; Ojeda-Magana et al., 2014; Martin-Sotoca et al., 2017). Therefore, further progress is needed in this area, especially in order to segment images containing multiple distinct populations of voxels.

### BIB- and FIB-SEM

Another approach that has recently been explored to obtain basically the same physical information as with X-ray CT consists of using broad- or focused ion beam scanning electron microscopy (BIB- or FIB-SEM). The ion beam can directly modify or “mill” a specimen surface, and this milling can be controlled with nanometer precision. By carefully controlling the energy and intensity of the ion beam, it is possible to perform very precise nano-machining to remove very thin layers of material, for example in a block of soil impregnated with resin. BIB milling produces cross-sections of a few mm^2^ to cm^2^, whereas FIB deals with surfaces that at most are a few hundred μm^2^. Once a new surface has been exposed, it can be imaged via SEM, at resolutions typically between 10 and 500 nm (Cantoni and Holzer, 2014). The sequence of images obtained in successive layers can be assembled into a 3D image, similar to those resulting from X-ray CT tomography, and subsequently segmented (Salzer et al., 2015; Liu et al., 2017). In the last few years, this approach has been used extensively to investigate the morphological characteristics of dolomite rocks, shales, and clays using BIB alone (Houben et al., 2013), a combination of BIB- and FIB-SEM (Hemes et al., 2015), or the joint use of micro-CT and FIB-SEM (Devarapalli et al., 2017). In soils, FIB-SEM presents a tremendous potential, but its use appears to have been limited so far to observations of microbially induced calcite precipitation in sandy soils (Li et al., 2017) and to obtain high-resolution images of the colonization of soil–root interfaces (Vidal et al., 2018).

### Soil Structure Versus Architecture

Early in the use of CT scanners to characterize the physical properties of soils, it became apparent that this technology afforded a convenient response to the age-old question of how to best quantify soil “structure,” this term being understood either as “the arrangement or organization of the particles in the soil” (Hillel, 2004), or, following Dexter (1988), as “the spatial heterogeneity of the different components or properties of soils.”

For many decades, the vast majority of the research on the topic has viewed soil structure as intimately linked with the fact that it is possible to fragment soils into distinct aggregates upon the application of mechanical stress (Rabot et al., 2018). Undoubtedly this perspective has its roots in the soil surveyors’ traditional poking of exposed soil profiles with knives, leading to the detachment of chunks of soils, called “aggregates,” whose size and shape is used to diagnose the types of pedogenetic processes that might have taken place at that location, to classify soils, and to evaluate their agronomic potential. Since the 1940s, an extensive body of literature has been devoted to the assessment of the stability of soil aggregates under a variety of operational conditions, for example under dry or wet sieving. As Young et al. (2001) point out, “the ease and seeming reproducibility of the many standard stability tests are the main drivers behind the prevalence of this type of research.”

A common criticism of the concept of aggregate in soils is that it is little more than an artifact. The hierarchical organization of aggregates, identified and described in detail by Tisdall and Oades (1982), suggests that the distribution of sizes of aggregates one obtains might depend on the amount of energy that is applied to take soils apart. This operational issue, discussed by Amézketa (1999), is particularly well illustrated by the experimental results of Díaz-Zorita et al. (2002), who show that the size of fragments obtained by sieving soils is inversely related to the mechanical stress applied. Hallett et al. (2013) also point out that breakdown of soils by dynamic or static mechanical loading yields different fragmentations of soil aggregates. This dependence of the aggregate size distribution on the operational conditions under which it is measured raises the question of whether aggregates exist in soils in their natural state (Young et al., 2001), calling into question the extensive literature that tries to analyze the influence of aggregate size on various processes, e.g., in terms of the sequestration of OM, the distribution of bacteria, a wide range of geochemical processes, or the release of greenhouse gasses (Ranjard and Richaume, 2001; Jasinska et al., 2006; Nunan et al., 2006; Razafimbelo et al., 2008; Goebel et al., 2009; Pallud et al., 2010; Chivenge et al., 2011; Masue-Slowey et al., 2011, 2013; Blaud et al., 2014; Rabbi et al., 2014, 2016; Ebrahimi and Or, 2015; Jiang et al., 2015; San José Martínez et al., 2015; Sheehy et al., 2015; Hausladen and Fendorf, 2017; Rillig et al., 2017; Zhao et al., 2017; Bocking and Blyth, 2018; Li et al., 2018), and explaining perhaps why some authors have failed to observe anticipated correlations between OM content and aggregation (Razafimbelo et al., 2013). Nevertheless, one might argue that this dependence problem can be alleviated somewhat by standardizing methods, and that, in any event, it does not particularly affect attempts to understand at a very local scale in soils the interactions between pore geometry, chemical composition, and microbial activity. As long as aggregates are viewed as chunks of soil that are convenient to manipulate because they do not fall apart too easily, e.g., when they are rotated on the stage of a CT scanner, and to the extent that no particular significance is associated with their external surfaces, which might just have been failure planes in some larger aggregate, no harm is done in using aggregates to gain insight into microscale processes, as various authors have done successfully (Remusat et al., 2012; Ananyeva et al., 2013; Kravchenko et al., 2013, 2015; Voltolini et al., 2017; Yu et al., 2017).

One could also consider that there is no problem either with repacking aggregates extracted from a soil, and trying to find out experimentally or through simulation how this now entirely artificial system behaves (e.g., Daly and Roose, 2014; Ebrahimi and Or, 2016). We are often forced by journals to use repacked soil columns in order to have actual replicates, and be able to calculate statistics, which some reviewers view as sacred and indispensable. However, it is entirely unclear at this point to what extent the conclusions that one reaches from this kind of exercise relate to the behavior of real soils, including the very soil from which the aggregates that are used originated. The reason for this has to do fundamentally with the absence of any theoretical framework or set of procedures to, as it were, put the pieces of the puzzle back together, once a soil has been disaggregated and its aggregates have been characterized, e.g., relative to their size distribution and individual geometries. In the process of disaggregating a soil sample, as long as no information is obtained about the geometry and topology of the interstices that may have existed originally between what eventually becomes distinct aggregates, there is no way practically to “reconstruct” the original soil, even for computational purpose, and in particular to guarantee that the pores between aggregates in the repacked system be similar in shape to those that existed originally^1^. One could draw parallels here with architecture (Letey, 1991; Baveye, 2006) or even with card games: Indeed, one cannot say anything about the size and shape of a house of cards after it has been torn down, simply by looking at the pile of cards that is left.

Aware of these obstacles already many years ago, a number of authors argued for a different way to approach the structure of soils. Dexter (1988), in a thorough review of the then available methodology in this field, recommends that preference be given to methods involving direct observation of structural features by scanning electron microscopy and by optical scanning of resin-impregnated sections and fracture surfaces. A few years later, Letey (1991) vents his frustration in the face of many failed attempts to link soil structure, defined in terms of aggregates, to functionality within the soil system. He suggests that instead of focusing on the solid components of soil structure, as had been the tradition for decades, one should emphasize instead the arrangement of voids, and the properties that these voids confer to soils, just as to describe a building, it is not primarily the shape of the bricks or stones that matters, or the thickness of the walls, but the size of the rooms and openings (windows, door frames). Reiterating these same messages, Young et al. (2001) argue that “an investigation of discrete aggregates or distributions of aggregates does not offer any spatial information. Functional traits of soil structure, at all scales, rely on the connectivity, tortuosity, and heterogeneity of pore space in 3D.” The same message is echoed in the recent thorough review of the literature by Rabot et al. (2018), who conclude that “although appealing, the aggregate perspective does not seem to be the most appropriate to link soil structure with soil functions and processes.” Because of the historically close connection between “soil structure” and aggregates, Young et al. (2001) propose to drop the expression of “soil structure” in favor of that, less history-laden, of “soil architecture.” This terminology has been routinely adopted since (e.g., Baveye, 2006; Lin et al., 2010; de Jonge et al., 2012; Lin, 2012; Bouckaert et al., 2013a,b; Cazelles et al., 2013; Helliwell et al., 2013; Kravchenko and Guber, 2017; San José Martínez et al., 2017) and will be used consistently in the following.

In principle, it is feasible to analyze this architecture by taking 2D images of sequences of thin sections in resin-impregnated blocks of undisturbed soil, and then using dedicated software to reconstruct from these images a full 3D picture of the geometry of soil pores. This tedious, time-consuming approach has been adopted with success by Cousin et al. (1996, 1999), Vogel (1997), and Vogel and Roth (2001). However, access to X-ray beams at various synchrotron facilities, and especially the availability of table-top X-ray CT scanners, have allowed the work in this area to experience a quantum leap around the turn of the century. The new technology has made it possible to obtain 3D images of the pore space in intact soil cores much more rapidly, and at resolutions that have gradually improved over time (Mooney, 2002; Rozenbaum et al., 2012; Bouckaert et al., 2013a; Calistru and Jitareanu, 2015; Rabot et al., 2015).

The gradual conceptual shift from the aggregate-based “structure” to the “architecture” of soils has been accompanied by a refocus of the discourse on the voids within this architecture, following in that respect the suggestion of Letey (1991). Another conceptual shift as well is occurring in that respect. Conditioned to think in terms of a traditional analogy between the pore space of soils and a bundle or network of capillaries, soil physicists used to be concerned about the size of “pores” in soils. It is clear from CT images that there are no identifiable pores in soils, and that the delineation of individual pores is necessarily somewhat subjective. Some authors have tried to make the concept of pore size distribution less arbitrary by using automatic algorithms to determine locally the radii of maximum balls that are fully inside the pore space. Partly because of the historical weight of the capillary analogy and partly with the help of these “inscribed balls” algorithms, pore size distributions are still being computed (e.g., Kuka et al., 2007; Papadopoulos et al., 2009; Ostadi et al., 2010; Bouckaert et al., 2013a; Peng et al., 2014; Houston et al., 2017; Meira Cassaro et al., 2017). Yet, clearly, researchers have increasingly turned in recent years to other approaches to describe quantitatively the make-up of soils. Indeed, a whole panoply of mathematical tools is now available, and is steadily expanded, to characterize a number of aspects of the pore space. These tools include various algorithms to calculate the tortuosity and connectivity of the pore space on the basis of grayscale or binary 3D CT images (Gommes et al., 2009; Houston et al., 2017; Meira Cassaro et al., 2017). Another approach to describe the pore space quantitatively is provided by the fundamental set of Minkowski functional measures (Lehmann et al., 2006; Vogel et al., 2010; Falconer et al., 2012). These functional measures comprise the volume, surface area, integral mean curvature, and the Euler-Poincaré characteristic (or topological measure Chi). Karsanina et al. (2015) propose another set of descriptors, including two-point probability functions, linear functions, and two-point cluster functions, and they used the first two in simulated annealing optimization procedures to reconstruct soil architecture artificially, based on original images of soil thin sections. For a number of years, fractal geometry was thought to be an ideal tool to characterize the inner space of soils, since according to the way the theory was interpreted, a single parameter, the fractal dimension, could inform about the make-up of soils over a range of scales. This vision of fractal geometry has since been confronted with the reality that a single dimension, whose value turns out to be itself scale- and resolution-dependent, does not suffice. As explained by Mandelbrot from the start, at least one other parameter, either the lacunarity (e.g., Pendleton et al., 2005; San José Martínez et al., 2017) or the succolarity (de Melo and Conci, 2013) is required to obtain an accurate description. Since the lacunarity (Pendleton et al., 2005) and likely also the succolarity are affected by the resolution of images, it is not clear at this point whether fractal geometry still offers much interest. Another approach that might (but has not yet) provide a solution to the resolution-dependence derives from the application of the theory of multifractal measures to CT images of soils (Lafond et al., 2012; Wang et al., 2016; Torre et al., 2018). From a different perspective, various authors have also tried to extract networks from 3D images of soils, to which graph theory principles can be applied to characterize the connectivity and topology of the pore space (Vogel and Roth, 2001; Perez-Reche et al., 2012).

### Can We “See” Water and Organic Matter in Soils?

Since the ultimate objective of the research reviewed here is to eventually be able to predict the activity of microorganisms in soils, for whom the presence of water and readily biodegradable OM is crucial, it is important to be able to detect in what portion of the pore space they are located. In this respect, researchers have been confronted with the difficulty that, typically, if one places a wet soil sample in a table-top X-ray CT scanner, the outcome is a 3D grayscale image characterized by a histogram with a single, broad peak that is not suitable at all to tease apart the water from the solids without resorting to arbitrary assumptions (Tracy et al., 2015), nor to identify in the solid phase the portion that corresponds to OM. So far, to avoid this obstacle, researchers have either shifted their attention toward artificial media, or they have worked with actual soils but under special conditions that allow the identification of water and OM.

In terms of artificial porous media, researchers have used glass beads (Culligan et al., 2006; Schaap et al., 2007) and coarse sands (Brusseau et al., 2007) to quantify the 3-dimensional distribution of water in the pore space. If one scans these systems under partially saturated conditions, as these researchers did, evidence suggests that it is not very difficult to locate air-water interfaces. Another way to proceed, made possible by the low reactivity of glass beads or sands compared to soils, is to increase the contrast between the attenuation of X-rays in the solid phase and the liquid phase by using a contrast agent that increases X-ray attenuation in the liquid. Although the results obtained with glass beads and sands are definitely interesting and probably applicable to coarse aquifer materials, it is not clear at this stage how they help us identify water and OM in actual soils, which as a rule tend to be tremendously more heterogeneous, and have much smaller pores. There is no real answer at this point to the question of how one can transition from glass beads to actual soils. This is a perennial problem, also faced by researchers who for a time carried out extensive work in the 1960s on the hysteresis of water retention in glass beads systems (Topp and Miller, 1966; Topp, 1971).

Another approach that can conceivably work in some soils, consists of scanning a soil sample when it is dry, and then re-scan it when it has been brought to the desired moisture content (e.g., Tracy et al., 2015). Comparison between the non-air phases in the “before” and “after” images yields the distribution of water in the system. In principle, this approach could work very well if the soil does not swell at all when its moisture content is increased. This apparently was the case in the experiments carried out by Tracy et al. (2015), who report that “no significant evidence of shrinkage was observed.” Yet the problem is that most soils in the world do shrink/swell to some extent (Garnier et al., 1997), including soils like those described by Radulovich et al. (1992) whose kaolinitic mineralogy one does not traditionally associate with this phenomenon. The question remains at this point of what is significant enough evidence of shrinkage or swelling in a soil sample to prevent this “subtraction” method to be used to visualize the distribution of water.

**FIGURE 2:**
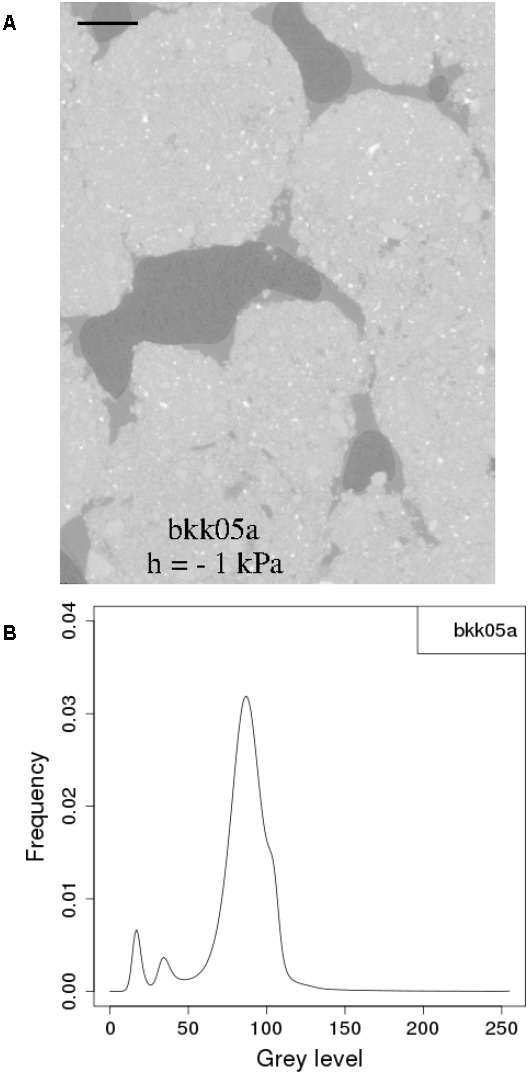
**(A)** two dimensional section of synchrotron X-ray computed tomography image of a soil cube equilibrated at –1 kPa and **(B)** histogram of the corresponding 3D SR-μCT image. In the tomographic sections, black is the air phase, dark gray is the water phase and light gray to white is the matrix phase. The scale bars represent 500 μm. (Reprinted with permission from Pot et al., 2015).

Yet another strategy is to carry out CT measurements on real soils under conditions where water and OM are not intimately mixed with the solid constituents at very fine scales. This approach has been adopted by a number of researchers in the last few years who worked on plant residues within soils (De Gryze et al., 2006; Negassa et al., 2015; Kravchenko et al., 2017) or attempted to directly visualize soil moisture (Carminati et al., 2008; Tippkötter et al., 2009; Pot et al., 2015). Working with a real clay-loam soil material near water saturation, Carminati et al. (2008) focused on the water that occupies part of the volume in the larger pores. They were able under these conditions to clearly observe pendular rings of water in images at a resolution close to 6 μm. Tippkötter et al. (2009) adopted a similar focus, in undisturbed soil samples, and were able with a table-top X-ray CT scanner to visualize the presence of water films coating the inner surfaces of meso- and macropores. Similarly, Pot et al. (2015), working with synchrotron X-rays, were able to generate CT images of repacked aggregates in whose histograms there was a good separation of voxels associated with the air, liquid, and solid phases (**Figure 2**).

A last approach that could work in principle to see the moisture in soils consists of adding various contrast agents to the water, to modify its X-ray attenuation (Van Loo et al., 2014). However, in practice, contrast agents need to diffuse sufficiently for the method to work, which again, in many cases, in undisturbed soil samples, might be operationally workable only to image the largest pores near saturation.

It might thus be tempting to look elsewhere for a possible answer. Indeed, over the last decade, the resolution associated with 3D nuclear magnetic resonance (NMR) micro-imaging has become steadily better. Lee and Lee (2017), for example, managed to obtain images of 1.2 mm by 1.2 mm columns of glass beads and crushed silica gels particles, respectively, with a spatial resolution of 46.875 μm, which is still coarser than the resolution of CT scanners for this column width, but is not as far from it as it used to be. As encouraging as these results are, however, NMR micro-imaging as currently implemented still suffers from a major obstacle when it comes to real soils, and therefore is not a real solution in that context. Because of the very powerful magnets that are used to generate the signal, only soils that do not contain paramagnetic elements can be imaged. Since many if not most soils contain some iron, at least, this limits tremendously the conditions under which NMR is a viable alternative to X-ray CT to generate 3D images. An alternative to NMR would be to use neutron computed tomography (Tumlinson et al., 2008) to observe the distribution of water in soils, but at this stage the resolution of images that can be generated is still relatively low, comparable to that obtainable with medical or table-top X-ray CT equipment 15 or 20 years ago (Perfect et al., 2014).

The best option to “see” water at this point, even though it has not been implemented very much of late, appears to be the use of dual energy X-rays in CT scanners. With gamma rays of two different energies, typically produced by ^241^Am and ^137^Cs sources, it has been possible for a while to simultaneous assess the moisture content and bulk density of soils (Soane, 1967; Hopmans and Dane, 1986; Biassusi et al., 1999), but the measurements are extremely slow, and their spatial resolution is low. Garnier et al. (1998) applied dual-energy synchrotron X-rays for the first time to soils, to assess rapid vertical soil density and water content changes in swelling soils during infiltration. Shortly thereafter, Rogasik et al. (1999) used a medical scanner that allowed them to scan silt loam subsoil samples at two energy levels (80 and 120 kV) to evaluate the distributions of water, air, and solids, as well as the voxel dry bulk density. The spatial resolution during scanning was 0.25 mm in the horizontal and 1 mm in the vertical direction, which was (and still is) standard for scanners routinely used in hospital settings. Since this work almost 20 years ago, there has been to our knowledge no application of dual-energy X-ray tomography to soils. Several table-top X-ray scanners currently commercialized offer the possibility to carry out dual-energy scanning sequentially on soil samples. The fact that nobody so far has reported on the use of this feature with table-top scanners suggests that polychromatic X-rays are not suitable for dual-energy scanning to work in the case of soil samples. Further research is needed to determine if with monochromatic X-ray beams, at synchrotron facilities, dual-energy scanning produces promising results.

**FIGURE 3:**
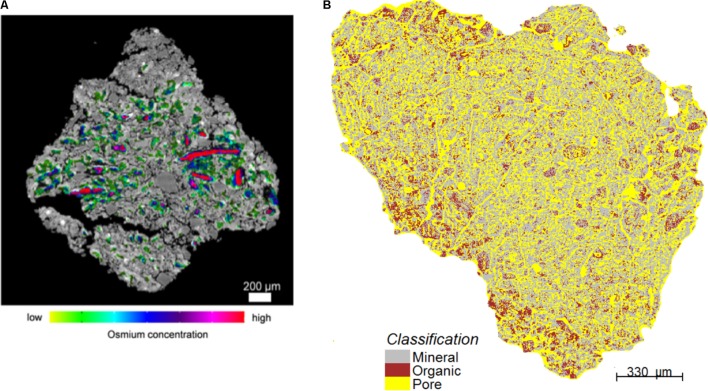
Illustrative two-dimensional spatial distributions of osmium-stained OM mapped onto the reconstructed image cross-sections of aggregates. In **(A)**, red colors are typically associated with particulate OM. Green colors reflect lower concentrations (Modified from Peth et al., 2014. Reprinted with permission). In **(B)**, the red patches correspond to OM (Modified from Rawlins et al., 2016. Reprinted with permission).

Part of the reason for the limited use of dual-energy scanning - and it would be true as well for attempts to scan soils rich in OM with dual-energy gamma-rays - is that until not too long ago, it would have been difficult to tease apart water from soil OM in CT images (Taina et al., 2008). The problem is not OM *per se*. Kettridge and Binley (2011)demonstrate that X-ray CT can image beautifully the structure of peat samples of various compositions. The difficulty has to do with the fact that at the high X-ray energies required to penetrate through soil materials, there is very little difference in X-ray attenuation between water and water-filled OM, whose peaks in grayscale image histograms are often not clearly distinguishable from a broad peak associated with mineral constituents. This problem was resolved, at least in part, in 2014, when Van Loo et al. (2014) tested 52 different chemical compounds. They perfused aqueous solutions saturated with the compounds through undisturbed soil samples under partial vacuum and found that 4 of these chemicals [phosphomolybdic acid (PMA), silver nitrate, lead nitrate and lead acetate] successfully enhance the X-ray attenuation contrast of OM relative to soil minerals and allow particulate organic matter (POM) to be easily detected. Peth et al. (2014) tried to take advantage of the fact that osmium has a marked absorption K-edge^2^ at a photon energy ∼74 keV. They exposed air-dry soil aggregates to a 25 w/w OsO_4_ solution for 48 h at room temperature in a closed vial under a fume hood (because of the very high toxicity of OsO_4_), and scanned these aggregates at a synchrotron facility below and above the absorption K-edge, respectively. Preliminary results, obtained by Peth et al. (2014) and Rawlins et al. (2016), suggest that this technique makes it possible to visualize the distribution of OM in soils, and to distinguish between POM and OM that is distributed more diffusely throughout the soil architecture (**Figure 3**). One promising approach to identify POM in CT images consists of building on both the attenuation, thus gray scale, values of the organic materials and on the spatial distribution patterns of POM grayscale values, which uniquely separate it from the rest of the soil solids (Kravchenko et al., 2014a). Indeed, even from a “naked eye” examination, POM often stands out on CT images due to much greater uniformity of its grayscale values. Kravchenko et al. (2014a) successfully used geostatistical parameters of POM fragments as indicators of the presence of POM in intact soil samples. This approach has advantages over POM identification via Os staining, since, unlike Os staining, CT scanning has minimal effect on soil microorganisms (Bouckaert et al., 2013b; Kravchenko et al., 2014b; Schmidt et al., 2015). Thus, the samples can be used for exploring the decomposition of the identified POM fragments in a sequence of initial CT scanning, incubation, and post-incubation CT scanning activities, as done by Kravchenko et al. (2015). However, as of now the process of POM identification using this approach is time consuming and requires a substantial user input.

### Sub-Resolution Pores

Another issue that, at the moment, is still awaiting a definite solution, is related to the soil pores having dimensions smaller than the resolution of CT images. From the mid-1980s to the early 2000s, the resolution of X-ray CT scanners accessible to soil scientists improved by about 3 orders of magnitude, from a few hundred microns to a fraction of a micron in the best of cases. However, no matter how small this resolution is, a portion of the pore space unavoidably remains invisible to scanners. The practical significance of sub-resolution pores depends strongly on the resolution of CT images, of course, but also, critically, on the type of soil considered. For a coarse sandy soil, it is possible that at a 20 or 30 μm resolution, only a minute portion of the total porosity would not be visible in thresholded CT images. Contrastedly, in other types of soil, the portion of sub-resolution pores can be substantial. In microaggregated tropical soils like those investigated by Sollins and Radulovich (1988), pores with a diameter less than 5 μm make up approximately 70% of the pore space, and are key to understanding the unique physical and chemical properties of these systems (e.g., Radulovich et al., 1992). In the silty soils investigated by Piccoli et al. (2017), 82% of the pores a dimensions smaller than the resolution of 30 μm at which the CT scans were made. In clayey or clay-loam soils, like those whose pore size distribution was determined via mercury intrusion porosimetry by Churchman and Payne (1983), a resolution of 10 μm would be too large to identify any pore at all, and it would be of paramount importance to be able to somehow characterize the sub-resolution porosity in some fashion.

In some very special cases, grayscale CT images, before any thresholding or segmentation is carried out, may contain some information about sub-resolution features. In porous media like sandstone samples or columns filled with glass beads, which consist solely of a homogeneous mineral phase, grayscale values intermediate between those of the pores and solids in CT images result from partial-volume effect if the solid particles are not porous. If, on the contrary, the solid particles are themselves porous, intermediate grayscale values are associated both with partial-volume effects and with sub-resolution pores, and blind application of a ternary segmentation approach, like that used by Scheibe et al. (2015), may yield meaningless results if interpreted solely as sub-resolution porosity. However, in these same simple systems, as long as partial-volume effects are negligible, grayscale values contain unambiguous information about sub-resolution pores, information that is lost entirely when the images are thresholded (Gommes et al., 2009; Baveye et al., 2010). To refine the analysis, insight obtained from images of these systems at different resolutions can be combined, as was done recently by various authors (Sok et al., 2010; Pini and Madonna, 2016; Shah et al., 2016) and applied to soils by others (Vogel et al., 2010; Dal Ferro et al., 2014), to try to get a better handle on predicting the properties of sub-resolution voids. Unfortunately, in real soils, in most cases of practical interest, both these single- and multiple-resolution approaches run into as yet insurmountable hurdles, related to the intimate, fine-scale mixing of minerals, OM, and water. The contribution of these different phases and constituents to the grayscale value of voxels in reconstructed CT images cannot at this stage be differentiated, and as a result, it is not possible in general to correlate this grayscale level with the porosity of the volume of soil to which it corresponds. It might be necessary to wait until tunable X-ray and gamma-ray scanners become routinely available, to resolve this issue.

### Moving From 3D to 4D: Dynamical Measurements

In order to get a dynamical picture of physical processes in soils, one needs to transition from 3D to 4D, the fourth dimension of course being time. In many disciplines outside soil science, this transition has captured the attention of researchers over the last few years, and very interesting results have been obtained, in particular for very coarse-textured porous media (Berg et al., 2013; Dobson et al., 2016). Yet, as far as soils are concerned, forays along these lines have been timid. At the mesoscale, very interesting work, starting already 25 years ago, describing how earthworm burrow systems evolve over time (Joschko et al., 1991; Capowiez et al., 1998), how the geometry of macropores in paddy soils evolves during soil shrinkage (Bottinelli et al., 2016), or how loamy soils are compacted during centrifugation (Schlüter et al., 2016). Also, various researchers have used MRI systems to monitor the infiltration of water in clay and coarse sandy loam columns (Amin et al., 1994, 1996; Preston et al., 2001; Votrubová et al., 2003), γ-ray CT equipment to quantify the swelling of vertisols over time (Biassusi et al., 1999), or neutron CT systems to investigate the dynamics of water flows in soil, especially in the rhizosphere (Badorreck et al., 2010; Perfect et al., 2014; Tötzke et al., 2017). However, virtually all of this work has been carried out at relatively low resolutions, at best of 15 μm but more often than not of several tens or even hundreds of microns.

At the micron scale sensu stricto, very little 4D work has been carried out so far. None of this research includes water movement, which is not very surprising, given the difficulties mentioned earlier concerning the detection of water at a sufficiently high resolution to be relevant to the microscale. Even under the various conditions where this detection is possible, water movement tends to be too fast to be monitored by X-ray CT, even at the fastest scanning times (of the order to 10–15 min, typically) available with table-top scanners. Ultrafast scanning techniques have been used recently with columns filled with gravel (Dobson et al., 2016), but similar research has yet to be conducted with soils. Because of these constraints, dynamic microscale measurements have been limited to situations that involve ice formation, or slow changes in the architecture of soils. Using a table-top X-ray CT scanner, Torrance et al. (2008) investigated the changes in structure and the redistribution of water to form ice lenses in saturated samples of an Aurora silt loam frost-susceptible soil that were thoroughly mixed to produce an initially homogeneous material, and of a Honeywood silt loam that was deliberately contaminated with motor oil. The soils were subjected to relatively rapid, downward freezing, with access to water at their base. The results indicate that CT can produce excellent images of the ice lens distribution within a frozen silt loam soil, the consolidation of soil between the ice lenses, and the effects of hydrocarbon contamination on ice formation. Also using X-ray CT in freezing soils, Starkloff et al. (2017) assessed the impact of a succession of freezing-thawing cycles on the pore network of a silty clay loam and a loamy sand topsoil. Also recently, Schlüter and Vogel (2016) quantified soil architecture turnover by labeling soil constituents in place with small garnet particles and tracking their fate in successive CT images. The particles adhere to pore boundaries at the beginning of the experiment but gradually change their position relative to the nearest pore as structure formation progresses and pores are destructed or newly formed.

### Modeling the Physics

Over the last 2 decades, a significant body of literature has been devoted to the mathematical modeling of water retention and transport within the complex geometry of soil pores, revealed with increasing resolution by X-ray CT scanners. The bulk of this literature has dealt with the development and application of the Lattice-Boltzmann method (Martys and Chen, 1996; Genty and Pot, 2013, 2014; Liu et al., 2016; Cruz et al., 2017; Zhou et al., 2018), but in recent years, other methods have also been adopted, based on finite element or finite difference schemes, or on geometric primitives.

Most of the work in this area has involved a number of variants of the Lattice-Boltzmann method, in which a fluid is viewed as a collection of fictitious particles that, alternatively, propagate from node to node on a regularly spaced grid (lattice mesh), then collide with the particles that end up on the same nodes. In the modeling of soils, the nodes correspond to the centers of voxels in 3D CT images. The method originates from a molecular description of a fluid and can directly incorporate physical terms stemming from a knowledge of the interaction between molecules. Hence, in principle, it keeps the cycle between the elaboration of a theory and the formulation of a corresponding numerical model short, which undoubtedly explains the enthusiasm it incited as soon as 3D CT images of soils became available. The key mathematical ingredient of the method is the probability $f_{q}\left(\overset{⇀}{r},t\right)$ of finding a particle at position $\overset{⇀}{r}$ in one of the microscopic directions envisaged within the lattice, at time *t*, where the subscript *q* is an index associated with a set of microscopic directions that are selected arbitrarily. Several discretizations of space can be used and are traditionally classified via the DnQm scheme, where “Dn” stands for “n dimensions” and “Qm” denotes “m speeds.” A common choice is *D*3*Q*19, in 3 dimensions and with 18 nearest neighbors considered around each node, described by the unit microscopic velocity vectors, ${\overset{⇀}{c}}_{q}$. In this case, the subscript *q* takes on 19 different values (including rest particles).

Classical Lattice-Boltzmann models applied to soils require CT images to be thresholded and assume that voxels associated with pores in binary 3-D images are totally permeable to water molecules, whereas those associated with solids are completely impermeable. Recognition of the significance of the sub-resolution pore space has prompted a sizeable number of researchers in the last couple of years to investigate ways to take this pore space into account explicitly in Lattice-Boltzmann models of water movement in soils, following Gao and Sharma (1994) and Freed (1998). The resulting “Gray” or “Partial-Bounce-Back” (PBB) Lattice-Boltzmann models consider that each voxel in the original, grayscale CT images has a given probability of penetration by water or solutes, and therefore a complementary probability that water or solute particles that penetrate the voxel eventually bounce back to their previous positions (e.g., Sukop and Thorne, 2006; Chen and Zhu, 2008; Han et al., 2008; Walsh et al., 2009; Jones and Feng, 2011; El Ganaoui et al., 2012; Gottardi et al., 2013; Walsh and Saar, 2013; Zalzale et al., 2013; Chen et al., 2014; Li et al., 2014; Yoshida and Hayashi, 2014; Ginzburg et al., 2015; Xie et al., 2015; Yehya et al., 2015; Apourvari and Arns, 2016; Bultreys et al., 2016; McDonald and Turner, 2016; Pereira, 2016; Zhang et al., 2016). In all this work, considerable advances have been made recently and a number of technical issues have been clarified (Ginzburg, 2016), yet a major experimental hurdle related to the evaluation of the penetrability of sub-resolution pores, which at this point remains an arbitrary parameter in the models. As discussed in detail by Baveye et al. (2017), this penetrability cannot be deduced simply from grayscale values in CT images, and there is no practical alternative yet available.

The Lattice-Boltzmann method has indisputably become the de-facto standard in pore-scale studies of water retention and transport in soils. One of the drawbacks of the method, however, is the very long (sometimes weeks-long) computational time it typically requires on personal computers. Open Lattice-Boltzmann environments like Palabos^3^ or OpenLB^4^ offer options to run the code on massively parallel computers and arrays of graphics processing units (GPUs), or to decompose the flow domain into manageable subportions, and researchers are increasingly resorting to these speeding techniques in applications of the Lattice-Boltzmann method to soils. Nevertheless, the relative slowness of the original method, unless one has access to large computer clusters, has encouraged various authors to explore other avenues to model soils at the microscale.

One of these avenues encompasses a technique called “smoothed particle hydrodynamics” (SPH) (e.g., Tartakovsky et al., 2007), which works by dividing a fluid into a set of discrete elements, referred to as particles. To these particles is associated a spatial distance (known as the “smoothing length”), over which their properties are “smoothed” by a kernel function. This means that the physical quantity of any particle can be obtained by summing the relevant properties of all the particles that lie within the range of the kernel.

Finite element or finite difference schemes are also among the alternative techniques that have been selected to solve Stokes’ equation within the pore space of soils (e.g., Liu et al., 2016). In a recent article, for example, Gerke et al. (2018) introduce the free software Finite-Difference Method Stokes Solver (FDMSS) that solves Stokes’ equation using a finite-difference method (FDM) directly on voxelized 3D pore geometries (i.e., without meshing). Based on explicit convergence studies, validation on sphere packings with analytically known permeabilities, and comparison against lattice-Boltzmann and other published FDM studies, these authors conclude that FDMSS provides a computationally efficient and accurate basis for single-phase pore-scale flow simulations. By implementing an efficient parallelization and code optimization scheme, permeability inferences can now be made from 3D images of up to 10^9^ voxels using modern desktop computers. Tracy et al. (2015) use another numerical technique, based on the SIMPLE (Semi-Implicit Method for Pressure-Linked Equations) algorithm (Patankar, 1980) to solve Stokes’ equation in the pore space of loamy sand and clayey loam soil samples. These authors did so with OpenFOAM, an open source Computational Fluid Dynamics toolbox.

A different path, beside the Lattice-Boltzmann method and the various numerical schemes just alluded to, consists of using a morphological model. Such a model involves the approximation of the soil pore space by a network of so-called volume primitives, i.e., simple geometric shapes that can be transformed at will and combined to represent more complex geometries (Monga et al., 2007; Ngom et al., 2012). One way to do so consists of using a geometrical algorithm based on Delaunay triangulation to determine the maximal balls of the pore space segmented from the 3D CT images. Maximal balls are defined as the balls included in the pore space but not included in any other ball included in the pore space. Then, a minimal set of maximal balls is extracted in order to obtain a compact representation of the pore space (Monga et al., 2009). The key advantage of the method is that it requires far fewer balls than voxels to cover the pore space, and one might hope in principle that this drastic simplification will carry over to the various processes (e.g., water retention, transport) that one wants to simulate. There is no guarantee in this respect, however, especially when models encompass not just physical processes but also (bio)chemical and microbiological ones. If the geometric primitives become too large, it may be necessary to divvy them up in smaller subcomponents in order to account adequately for the spatial heterogeneity exhibited by chemical and microbial processes in soils. The added computational time that would result from this division might very well negate the speeding up that theoretically results from the scheme.

**FIGURE 4:**
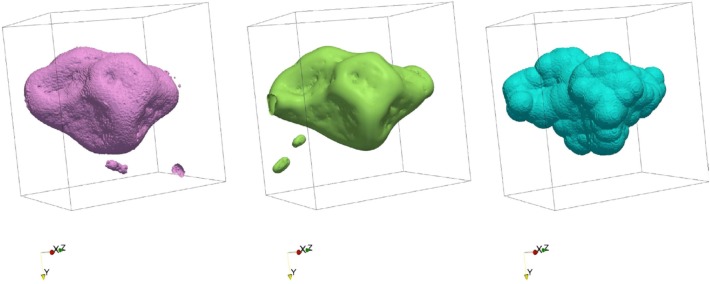
Air-water interface surfaces of a region of interest (region p1bkk04a) in a silt-loam soil, measured via synchrotron X-ray computed tomography **(left)**, predicted using the Lattice-Boltzmann method **(center)**, and predicted by the geometric primitive-based model MOSAIC **(right)** (Modified from Pot et al., 2015. Reprinted with permission).

Since there are different ways to simulate the retention and transport of water in soil pores, one might ask which of these methods performs best. The intercomparison of models (e.g., Yang et al., 2016) provides some general idea of the agreement, or lack thereof, among the models, but clearly, benchmarking model predictions against actual experimental data is by far the most desirable approach. At this juncture, since dynamic data about the movement of water (or other liquid phases, e.g., NAPLs) are not (yet) readily available, direct comparison with experimental data is feasible only for water retention in the pore space. Pot et al. (2015) carried out the only such comparison to date, on the basis of quantitative data of the distribution of water and air in soil samples constituted of repacked aggregates, equilibrated at three matric potentials (-0.5, -1, and -2 kPa). The phase distribution data were derived from synchrotron X-ray CT images at a resolution of 4.6 μm. Water distribution was simulated by a two-phase Lattice-Boltzmann model (LBM) and a morphological model (MOSAIC). Results indicate that the lattice-Boltzmann model is able to predict remarkably well the location of air–water interfaces (**Figure 4**). When one lifts the assumption, motivated by capillary theory, that a pore can drain only if a connecting pore is already full of air, MOSAIC gives an acceptable approximation of the observed air–water interfaces (**Figure 4**). However, discretization of pores as geometrical primitives causes interfaces predicted by MOSAIC to have non-physical bulbous shapes. Nevertheless, given the huge difference in computing time required to run these two models (minutes for MOSAIC versus tens of hours for Lattice-Boltzmann), Pot et al. (2015) recommend that further research be carried out on the development of both modeling approach, in parallel. One might argue that the same recommendation applies to other numerical schemes as well.

### Visual Summary of the Status of the Physical Front

**FIGURE 5:**
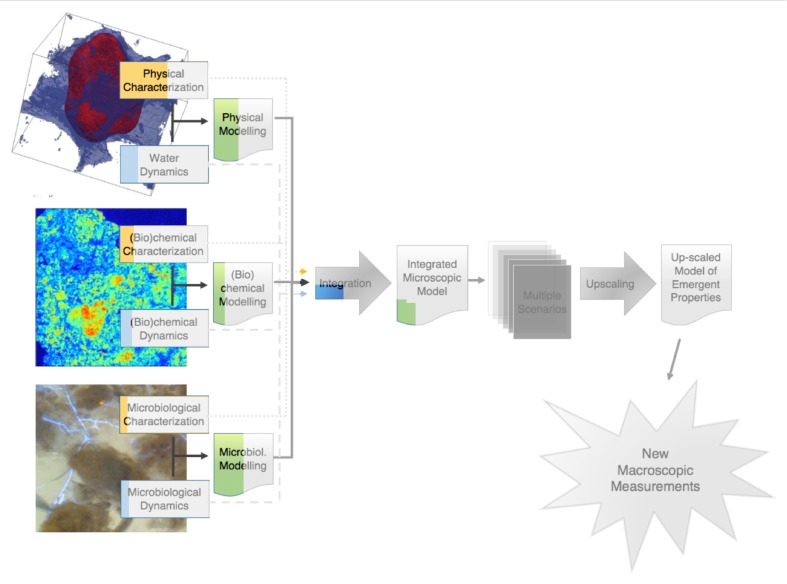
Visual assessment of the level of progress on the various steps in the research on the emergent properties of soils. The un-shaded parts (relative to the schematic diagram of **Figure 1**) correspond to the authors’ estimate of the progress achieved to date on each step. The shaded portion of the diagram still largely remain to be tackled.

Now that we have covered in some detail the progress achieved to date in the description and modeling of the physics of soils, it is probably a good idea, and a nice way to summarize things, to go back to the schematic diagram of **Figure 1**, and, with it, attempt to represent visually where we are at the moment. In **Figure 5**, this is done by shading in the diagram of **Figure 1** the parts that correspond to work yet to be carried out. Admittedly, this is a subjective exercise, and different researchers, depending on how pessimistic or optimistic they are, may come up with contrasting evaluations. Yet, based on the detailed account provided above, the depiction in **Figure 5** of the status of the physical front seems reasonable.

In terms of the physics, **Figure 5** summarizes visually the conclusion that work is relatively well advanced. Certainly, some areas require further research. Among others, the sub-resolution porosity of soils needs to be better apprehended. Yet, overall, significant progress has already been achieved on the experimental side. This is true to a lesser extent insofar as the dynamics of water (and other liquid phases) is concerned, due to difficulties in measuring changes in water content over sufficiently short times. In terms of modeling of the physics, some success has been achieved in the past decade, but there is room for improvement, in particular relative to the speed of computations.

## The (Bio)Chemical Picture

### Limited 3D Microscale Measurements

Physical information is not sufficient to characterize microenvironments. Information about the chemistry, and in particular about the nature of reactive surfaces (e.g., Kotani-Tanoi et al., 2007) and of dissolved or adsorbed (bio)chemical species, is also important. Therefore, to complement the information available about the geometry and topology of the pore space in soils with similar information about the chemical properties, one should ideally be able, as a start, to measure in 3D the chemical composition of soils. A logical and relatively simple option in this respect, in principle (Egan et al., 2015), would be to take advantage systematically of the X-ray absorption K edge of all the elements we might be interested in, in a manner similar to the determination of the distribution of soil OM by adsorption of osmium (Peth et al., 2014). Unfortunately, the situation with osmium is somewhat unique. First, Os happens to have its K edge at 73.87 keV, in the middle of the range of energies that is typically required to deal with soil samples of a volume larger than a few cm^3^. In the literature so far, that range has extended from 30 keV for synchrotron X-rays (Pot et al., 2015) up to nominally 225 keV for polychromatic X-rays (Houston et al., 2013b). Practically, the low end of this energy range means that, in soil samples of a reasonably large size, it is not possible to detect elements with an atomic number lower than 51 in the periodic table, i.e., before antimony (Sb), which has an X-ray absorption K edge at 30.49 keV (Bearden and Burr, 1967). This constraint entails that only a few elements, like I, Cs, Ba, Hg, Tl, and Pb, can be mapped in 3D in soil samples, but even then, yet another condition has to be met, namely that these elements be present in soils in such high concentration that they affect appreciably the level of X-ray absorption in the voxels where they are located. In soils containing only trace-level concentrations of these elements, voxel grayscale values would be unlikely to differ much, if at all, immediately below and above the various K edges. This means in effect that, naturally or artificially, soils would have to be heavily laden, at least locally, with these elements for their spatial distribution to be detectable. For example, to be able to use the X-ray absorption K-edge of Cs to detect the distribution and movement of water in fine sand samples, Willson et al. (2012) had to use 10% (by mass) CsCl solutions. Similarly, to detect the transport of CaI_2_ solutions within small sand columns using the K-edge of iodine, Shokri (2014) had to use concentrated (5% by mass) CaI_2_ solutions. Altman et al. (2005) submerged soil aggregates in a 507 g L^-1^ CsCl solution in order to saturate the exchange complex with Cs, prior to scanning samples above and below the Cs K edge. Similarly, Keck et al. (2017) used a 0.3 mol L^-1^ BaCl_2_ solution to assess the distribution of cation adsorption sites in four undisturbed soils, three of which have a very high clay content (53.9–79.9% clay). In both Altman et al.’s (2005) and Keck et al.’s (2017) cases, the clay content of their soils makes one wonder whether the amount of CsCl and BaCl_2_, respectively, needed for complete saturation of the exchange complex with Cs+ and Ba2+, which appears necessary to detect significant differences in X-ray attenuation, may have also caused, respectively, a dispersion of clay particles or a shrinking of clayey aggregates, and therefore, changes in the scanned images that could have been misinterpreted.

Another option for the 3D determination of soil composition is X-ray fluorescence. It is far less constraining in terms of the elements it can map (elements starting with Na, atomic number 11, are possible candidates), but unfortunately it suffers from a similar limitation on the size of soil samples that can be analyzed. The principle of the method is simple. When an atom is irradiated with X- rays of sufficient energy, it ejects an inner orbital electron. An electron from higher orbitals then falls to fill the vacancy in the lower energy state, resulting in the release of a fluorescent X-ray. The energy of the fluorescent X-ray given off is characteristic of the energy difference between the two orbital energy levels, which is specific to each element, while the intensity of the emitted X-rays is related to the elemental abundance in the sample being analyzed. In X-ray fluorescence tomography systems (Bleuet et al., 2010), also called confocal XRF scanners (Patterson et al., 2010; Lühl et al., 2013) or spectrometers (Smolek et al., 2012), the presence of focusing optic both on the path of the incoming X-ray beam and between the sample and the detector allows the 3D elemental profiling of the sample, provided the travel path of fluorescence X-rays within the sample, once they are produced, is not too long, so that their absorption is minimized. This re-absorption of X-rays limits the 3D measurements to minute samples, extending to at most a few mm in any direction. In that context, McIntosh et al. (2015) used 3D micro X-ray fluorescence spectroscopy to determine non-destructively the elemental composition of minute aggregates of a plutonium-contaminated soil, within which they could identify distinct 30 μm-size Pu particles with a limit of detection <15 ng.

### Many 2D Measurements Are Feasible

Until direct 3D mapping of the chemical properties of soils becomes technically feasible and more accessible, an alternative approach to obtain 3-dimensional chemical information about soils at the microscale is to carry out the same procedure used a couple of decades ago by Cousin et al. (1996, 1999), Vogel (1997), and Vogel and Roth (2001), to obtain insight into the physical properties of soils. The idea is to perform multiple cuts through soil samples, analyze in turn the (bio)chemical make-up of each exposed surface within the soil, then, using an interpolation technique, generate a 3-D picture from the data associated with the various surfaces. This procedure is routinely used for biological samples, such as human tissues, in which the serial removal of layers can be carried out easily, either by using a traditional microtome or a cryo-ultramicrotome, or via ion-beam ablation.

**FIGURE 6:**
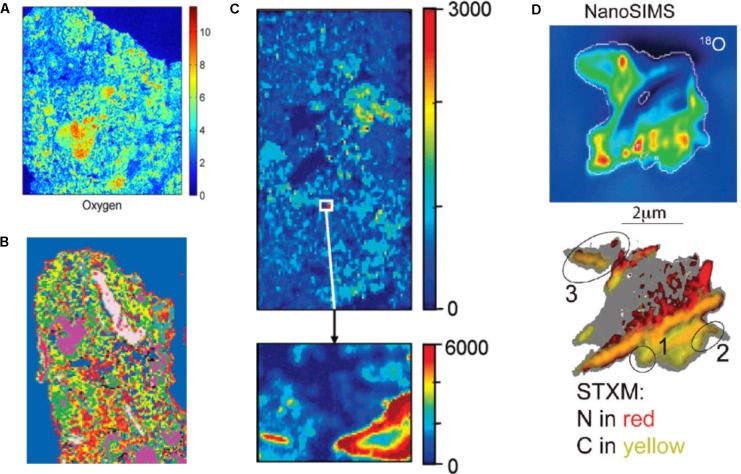
Illustrative examples of various chemical measurements that are now routinely carried out on 2D cross-sections through soil samples: **(A)** SEM-EDX mapping of the distribution of oxygen in a section through a calcareous soil from Scotland. The intensity of the color indicates the concentration of oxygen (Adapted from Hapca et al., 2015). **(B)** Cluster map showing the large heterogeneity of carbon forms within a soil micro-assemblage from Nandi Forest (Kenya) determined by NEXAFS in combination with scanning transmission X-ray microscopy (STXM). The field of view is 5.4 μmm by 7.5 μm (adapted from Lehmann et al., 2008. Reprinted with permission). **(C)** Synchrotron-based μXRF maps of Cu in a calcareous soil vineyard soil from Burgundy (France). The large map on top (2 by 4 cm) was obtained with a 0.3-mm spot size, the small map at the bottom (1.5 by 1 mm) with a 20-μm spot size. The color, from blue to red, is correlated with the Cu concentration (Adapted from Jacobson et al., 2007. Reprinted with permission). **(D)** NanoSIMS map of ^18^O in a soil aggregate (top) and C and N map of the same aggregate obtained by STXM (bottom). NEXAFS spectra were obtained in the three circled regions, whereas in the gray zones the sample was too thick to get NEXAFS spectra or was free of OM (Reprinted with permission from Remusat et al. (2012). Copyright [2012] American Chemical Society).

To these exposed surfaces, it is now possible to apply a panoply of different spectroscopic techniques, a luxury that not too long ago, researchers would not even have dreamt of. Indeed, until the mid-1990s, beside the standard bulk analytical methods, requiring a sizeable sample of soils and therefore precluding microscale analysis, the only method that was available to researchers to determine local (bio)chemical properties of soils was based on energy dispersive X-ray spectroscopy, either in the so-called “electron microprobe” or SEM-EDX equipment, or on electron energy loss spectroscopy (EELS) (Villemin et al., 1995). The first two of these instruments, in scanner mode, can in principle produce elemental maps like that of **Figure 6A**. Starting in the mid- to late 1990s, synchrotron facilities around the world began offering soil scientists the opportunity to run various types of analyses, including X-ray absorption near-edge structure (XANES) and near-edge X-ray absorption fine structure (NEXAFS) spectroscopies, which rapidly became popular because of the very useful information it is able to provide on the molecular environment of atoms, and therefore on element speciation (Prietzel et al., 2003; Schumacher et al., 2005; Solomon et al., 2005, 2012; Kinyangi et al., 2006; Christl and Kretzschmar, 2007; Wan et al., 2007; Strawn and Baker, 2009; Hesterberg et al., 2011; Milne et al., 2011; Jassogne et al., 2012; Kopittke et al., 2017). In most cases, the target of interest in this type of analysis is extremely minute in extent, a most a few μm^2^, but occasionally researchers have attempted to map properties over a slightly larger area, among other things to try to assess the heterogeneity of the composition of OM in soils (**Figure 6B**). Another synchrotron-based technique that has been used to some extent to obtain elemental maps involves X-ray micro-fluorescence (Hitchcock et al., 2004; Jacobson et al., 2007; Jassogne et al., 2012), which unfortunately does not provide information about speciation, but has the advantage that it can cover bigger surface areas (**Figure 6C**). Several other spectroscopic methods provide molecular- to micro-scale distributions of elements and isotopes and thus soil properties. Among them, the most commonly applied to soils in recent years is dynamic nanoscale secondary ion mass spectroscopy (NanoSIMS, **Figure 6D**). It uses a high-energy beam of ions (either Cs^+^ or O^-^) to eject secondary ions from a sample surface, which are then analyzed using a mass spectrometer, at a very high spatial resolution typically of the order of 100 nm for soil samples (Herrmann et al., 2007; Mueller et al., 2012, 2013, 2017). Slightly larger areas can be sampled with Static- or Time-of-flight Secondary Ion Mass Spectroscopy (Static SIMS or TofSIMS), which can target ions and small molecular fragments (Watrous and Dorrestein, 2011; Cerqueira et al., 2015; Worrich et al., 2017). Other spectroscopic methods, also working at spatial scales slightly larger than that of individual cells include Laser desorption/ionization (LDI), laser ablation inductively coupled plasma (LA-ICP), matrix-assisted laser desorption/ionization (MALDI) and desorption electrospray ionization (DESI) spectroscopies (Watrous and Dorrestein, 2011).

The fact that several of these techniques involve synchrotron X-ray beams, which are generally in extremely high demand, may explain why until now, their use has resulted in extremely few actual property maps of soil surfaces larger than microaggregates. Normally, experimentalists get “beam time” of 48–72 h at the most, during which it may be difficult to do a full scan of a thin section, for example. Due to the growing number of available instruments (soon to reach 50 worldwide), the situation for the use of NanoSIMS is improving, but access to all of them is highly coveted by researchers in many fields and only a hand full of laboratories routinely analyze soils. Fortunately, things might be getting better relatively soon in terms of NEXAFS since various groups of researchers (Peth et al., 2008; Müller et al., 2014; Kühl et al., 2016) have recently developed laser-based, benchtop-scale NEXAFS instruments, one of which is now commercially available. The results obtained to date, including on soil clays and OM, are very promising (Gleber et al., 2011; Sedlmair et al., 2012).

As exciting as the use of these various spectroscopic types of equipment might be, they afford measurements only of the concentration of various elements or their speciation, but not at all of the physico-chemical conditions in which specific (bio)chemical species are located. In particular, one would absolutely need information about the pH or redox potential locally in a porous medium, among other “thermodynamic” variables, to have a full picture of what is going on. Unfortunately, once a block of soil has been impregnated with resin and has cured, none of these variables is accessible any more. If somehow, one could cut through a soil, and obtain a relatively flat surface in the process, without having to solidify the soil and denature it in any way, it would be possible to obtain information on pH and redox potential through the application of microelectrodes, microsensor probes or planar optodes (Pedersen et al., 2015; Rubol et al., 2016; Keiluweit et al., 2018; Wanzek et al., 2018). Other gel-based approaches such as diffusive gradients in thin films (DGT, Santner et al., 2015) could be used to obtain 2D maps of the distribution of labile chemical species. Zymography is a methodology similar to “optodes” in that a planar membrane is brought to contact with exposed soil to measure the activity of various enzymes (Spohn et al., 2013; Razavi et al., 2016). The development of new sensors is a very active field, offering perhaps interesting opportunities for microscale soil characterization in a few years.

### Transitioning From 2D to 3D

Since many 2D measurements can be carried out on cuts through soil samples, it is feasible under certain circumstances to produce from them a 3D image of the soil (bio)chemical characteristics. As with anything one does, it is useful to inquire whether this step is absolutely required. If methods were readily available to provide us with 3D images of the (bio)chemical composition of soils, we would not necessarily ask the question of why we need 3D images in the first place, but since the best we can get experimentally is 2D images of cuts through soils, it is worthwhile asking ourselves whether we really need to go through the added effort of the transition to 3D. Some researchers may be interested mostly in the relative distribution of chemical elements with respect to the pore system, to evaluate local gradients and accessibility of substrate for soil biota. They may consider that information in this respect can be obtained by single 2D slices of chemical maps that are projected on the 3D pore structure. From this standpoint, serial sectioning and interpolation are not necessary. A different perspective on the question, held for example by Hapca et al. (2011, 2015) is that, just like the degree of connectivity or tortuosity of the pore space in 2-dimensional cuts through a soil are generally different than in 3-dimensions, the spatial characteristics of the chemical make-up of soils, the distribution and local concentration gradients of targeted (bio)chemical compounds, also need to be estimated in 3D if one is to understand their influence on microbial processes. Experience will show in the future which one of these two perspectives is most conducive to progress.

Nevertheless, to obtain 3D information on (bio)chemical properties, the process of interpolation between 2D maps is complicated by the fact that cutting through soils is not as straightforward as it may seem. In mineral soils, the frequent presence of dense constituents reduces the range of techniques that can be used to cut or scrape away successive layers with minimal disturbance. Particularly when operating microtomes, the presence of constituents with markedly different densities often causes blades to deviate from their set course, so that eventually the exposed surfaces are not perfectly flat. Because of that, the correspondence of 2-D chemical or microbiological maps with the physical information obtained via computed tomography is likely to be poor, unless artifacts generated during soil cutting are accounted for.

**FIGURE 7:**
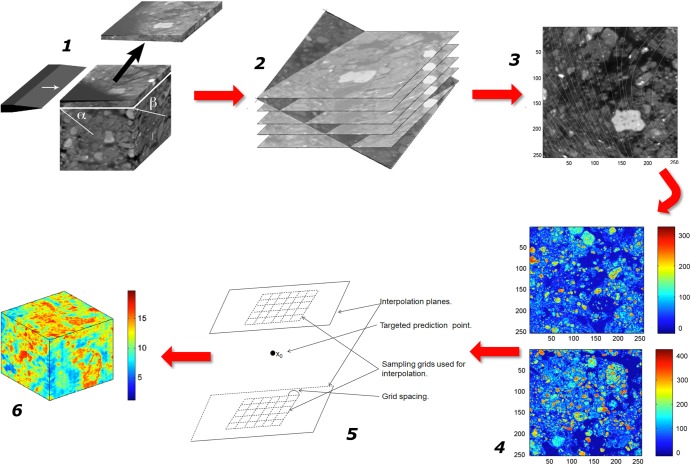
Schematic diagram of the successive steps in the 2D–3D interpolation method proposed by Hapca et al. (2011, 2015). **(1)** Illustration of a typical method to isolate a layer from a soil cube, with a microtome blade. The cut may be at angles α and β, respectively, with the *x*–*y* plane in the *x* and *y* directions, respectively, resulting in layer surfaces that are not strictly parallel to each other, **(2)** rotation of the chemical analysis plane within the 3D CT image, **(3)** Reconstituted CT image of the soil surface. The dotted lines correspond to the limits of the different masks applied to the successive layers during the zonation process, **(4)** Spatial distribution, measured with SEM–EDX, of silicon in the top and bottom soil surfaces of an individual slice through the soil sample, **(5)** schematic representation of the interpolation layers and the corresponding sampling grids for the selection of the interpolation points, **(6)** 3D prediction of the silicon distribution in soil sample.

Therefore, the first step in any attempt to simultaneously evaluate in 3D the physical, chemical, and biological characteristics of soil samples is to find a way to correct for any distortion that may occur when cutting or grinding down soil samples to successively expose surfaces on which 2-D chemical mapping is carried out, and to geo-reference these 2-D maps within the geometry of the soil solid phase, determined via X-ray computed tomography. A practical, automated procedure to accomplish these tasks has been developed by Hapca et al. (2011). This procedure, depicted in **Figure 7**, involves three successive steps, namely the reconstitution of the physical structure of a given soil layer surface, the alignment of the chemical maps with the reconstituted soil surface image, and finally the 3D alignment of the 2D chemical maps with the internal structure of the soil cube. Once this alignment is carried out satisfactorily, one can proceed to a statistical interpolation between successive geo-referenced 2D planes. Hapca et al. (2015) suggested that, for this interpolation, the 3D information produced via X-ray CT could be used as a guide. They proposed a method based on a regression tree method and ordinary kriging applied to residuals, and used it to predict the 3D spatial distribution of carbon, silicon, iron, and oxygen at the microscale. The spatial correlation between the X-ray grayscale intensities and the chemical maps made it possible to use a regression-tree model as an initial step to predict the 3D chemical composition. For chemical elements, e.g., iron, that have high attenuation and are sparsely distributed in a soil sample, the regression-tree model provides a good prediction, explaining as much as 90% of the variability in some of the data. However, for chemical elements with lower attenuation coefficients that are more homogenously distributed, such as carbon, silicon, or oxygen, the additional kriging of the regression tree residuals improved significantly the prediction with an increase in the *R*^2^ value from 0.221 to 0.324 for carbon, 0.312 to 0.423 for silicon, and 0.218 to 0.374 for oxygen, respectively. In principle, this method could be used for any (bio)chemical parameter that can be mapped on 2D cuts.

### Dynamical Picture

Given the need to be able to work with 2D cuts through soils, e.g., by impregnating soils with resin and cutting through the resulting blocks in one way or another, to carry out measurements of (bio)chemical characteristics, it should come as no surprise that dynamical measurements *at the microscale* have been so far, and may remain for some time, impossible to achieve (Blaser et al., 2016). Of course, dynamical measurements of properties as they emerge at the macroscopic scale can be achieved relatively easily, for example breakthrough curves in column experiments serving as evidence of chemical transport. To some extent and for specific purposes, such macroscopic properties may suffice, but experience has shown time and again that in an of themselves, these macroscopic observations are not adequate to assess the soundness of microscale models of (bio)chemical dynamics.

Much of the energy, nutrient and information (signal molecules) flows in soil occur in aqueous phase. A broad range of organic and inorganic molecular forms are released into solution, taken up and metabolized, transformed by enzymes or immobilized on surfaces continuously. These constitute the most dynamic portion of the microbial environment. Although much research has been carried out on the soluble OM of soil (e.g., Rousk and Jones, 2010; Lerch et al., 2011), the scale at which the measurements have been made is inappropriate for understanding how the flows of energy, nutrients and information vary throughout the soil pore network; only an average value is obtained. Furthermore, extraction methods tend to introduce biases related to the choice of extractant and the disruption of the physical architecture of soil may release molecules not previously in solution (Inselsbacher et al., 2014). The use of miniaturized sampling devices (millimeter scale) such as microdialysis probes or micro-suction-cups offer the possibility of detecting gradients in the soil solution at scales that are getting closer to those relevant to microbial communities (Inselsbacher et al., 2014; Oburger and Schmidt, 2016), particularly in the rhizosphere where gradients are stronger. With microdialysis the soil solution is sampled by passive diffusion and is therefore likely to better reflect the soluble environment perceived by microbial communities. Recent research has shown that there are significant differences in the size and composition of soluble organic and inorganic N pools measured by microdialysis and those measured by the traditional extraction methods (Inselsbacher et al., 2014). The small size of the probes makes it possible to locate them precisely in soil samples using micro-CT imaging and to map diffusive fluxes in real time (Brackin et al., 2017).

### Numerical Modeling

Over the last decade, a very significant amount of work has been devoted by geochemists and environmental engineers to the development of computer models able to describe the fate of a number of chemical species of interest in porous and fractured media (Tartakovsky et al., 2007; Valocchi, 2012; Steefel et al., 2013; Yoon et al., 2015). These models typically combine a transport component with a chemical speciation algorithm. Chemical transport is described using a variety of approaches, including the Lattice-Boltzmann method, smooth particle hydrodynamics (Tartakovsky et al., 2007), hybrid Lattice-Boltzmann-direct numerical simulation (DNS) (Yoon et al., 2012), and pore network models (Li et al., 2006). In terms of the chemical reactions that the speciation algorithms describe, as Iliev et al. (2017) accurately point out, many models focus on chemical reactions occurring in solution, with only a few models dealing with reactions controlled by the reactivity of the surfaces, like the dissolution of mineral phases. This bias makes sense for the type of systems researchers have been trying to describe, namely aquifer materials, calcareous formations, sandstones, or simply laboratory set-ups filled with glass beads. In that general context, one of the key predictions of these models has been that under a wide range of situations, macroscopic-scale descriptions with “effective” (i.e., volume-averaged) parameters do not account adequately for model predictions when non-linear reactive transport processes are associated with highly localized chemical reactions and incomplete mixing within the porous medium (Li et al., 2007a,b,c; Battiato et al., 2011; Steefel et al., 2013).

The different features of current microscale geochemical models of reactive transport probably explain to a large extent why none of them has been used so far to describe soil processes at the microscale. Given the extremely high specific surface area, and the significant surface reactivity of many soils, in addition to the fact that the reactions that take place in soils are complicated by the presence of very heterogeneous OM, the speciation portion of typical microscale geochemical models would have to be entirely overhauled before it could be applied to soils. Nobody, as far as we are aware, seems to have launched into this work yet. In addition, even if someone had done that work, model predictions could not at the moment be compared with actual microscale measurements at this stage, as discussed in detail in the previous two subsections.

### Visual Summary of the Status of the (Bio)chemical Front

If we try to summarize graphically the state-of-the-art of the (bio)chemical characterization and modeling of soils at the microscale, it is clear that work in this area is far less advanced than on the physical front (**Figure 5**). Measurements of static features or of the dynamics of (bio)chemical species are still very limited. Some of this scarcity of data can, however, be addressed. There is indeed a great potential to generate many static, 2D data, using a wide array of experimental methods, and to extrapolate them to three dimensions. Modeling frameworks are available to describe the transport of reactive chemical species in porous or fractured media, but they would need to be modified substantially before they could be applied to soils, and that significant effort has not taken place yet.

## The Microbiological Scene

### 3D Microscale Distribution of Microorganisms: Absence of Direct Data

To complement the 3D data related to the geometry and topology of soil pores, as well as 3D data about the (bio)chemical properties of soils, generated by interpolation among 2D pictures, it would be ideal if detailed 3D information could be obtained about the distribution of microorganisms in soils. Such information can be readily obtained in the case of wood, at least for fungi (Van den Bulcke et al., 2009). But unfortunately, for exactly the same reasons that hinder the direct 3D determination of the distribution of OM in soils, it has proven impossible so far to quantify the spatial heterogeneity of the distribution of microorganisms *in actual soils*.

When direct 3D measurements of biomass distribution in porous media have been obtained, it has so far always been under conditions that bear little similarity to real soils. Lilje et al. (2013) describe the development of a culture system and staining protocol they have used to obtain 3D quantitative data of filamentous and zoosporic soil fungi in an artificial matrix that was “developed to simulate the particulate nature of soil.” This artificial matrix consists of 500–900 μm diameter X-ray translucent polystyrene beads, which might be morphologically similar, to some extent, to coarse sand particles, but would likely have very different surface and hydration properties than typically highly heterogeneous soils. In many ways, the same comment pertains to the use of nuclear resonance imaging to detect “biofilms” in systems composed of polystyrene beads (Vogt et al., 2013). Sanderlin et al. (2013) pioneered the use of a very promising low-field magnetic resonance system to visualize the 3D distribution of biofilms in glass beads and sand particles, whereas a number of other authors used X-ray tomography to assess the distribution of biofilms in systems of glass beads (Davit et al., 2011; Iltis et al., 2011; Peszynska et al., 2016) or 2.5 mm-diameter Nafion pellets (Carrel et al., 2017). In all these cases, the properties and geometry of the systems investigated are drastically different from those of actual soils, which are generally characterized by a spatially dispersed- rather than concentrated biomass, and it is not clear at all at this stage how the transition from artificial media to actual soils will be made.

Since *direct* methods are lacking to quantify the 3D microscale distribution of bacteria in whole soil samples in one go, a number of authors have developed sampling techniques to obtain 3D information in other ways. Dechesne et al. (2003) developed such a technique and tested it in repacked soil columns. Their approach consists of a number of steps. The soil is first micro-sampled within several small subunit volumes of roughly the same volume (minimum sample side: length of 50 μm) within the columns, and these microsamples are subsequently tested for the presence or absence of targeted microorganisms, which in the original study were two bacterial strains but could equally easily have been archaea or fungi. A subsequent statistical analysis involves a comparison of experimental sampling data with data expected from limited sampling of numerous theoretical spatial distributions. Since the exact spatial location of the microsamples was not determined by Dechesne et al. (2003), they could identify only which *statistical* distributions of patches occupied by bacteria were possible within their sample. However, now that with CT, it might be possible to geolocalize small subsamples within soil columns, a similar approach could now be used to determine spatial distributions of various microorganisms as well, albeit at a relatively low resolution. An implementation of this approach is reported by Kravchenko et al. (2014b). In that work, ≈5 mm-sized soil fragments (referred to as macro-aggregates) were subjected to CT scanning, which provided information on pore architecture. Scanning was followed by cutting the macro-aggregates into geolocalized subsections, that is, the position of each subsection on the CT images was determined. Then, microbial community analyses of each geolocalized subsection via 16S rRNA pyrosequencing was conducted, enabling exploration of associations between presence of certain groups of microorganisms and abundances of soil pores of different sizes.

### Scarce Data on 2D Microscale Distribution of Microorganisms

**FIGURE 8:**
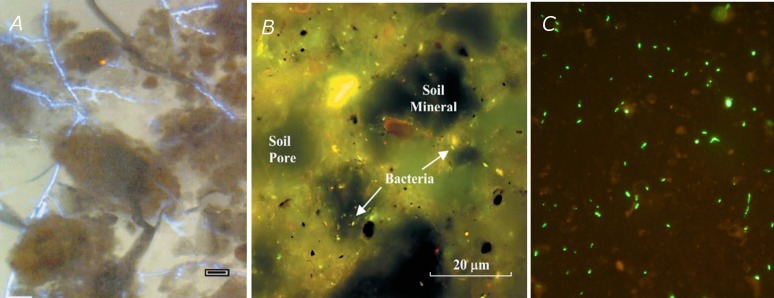
Examples of experimentally determined microbial distribution in soils: **(A)** microscopic image of hyphae of the fungus *Rhizoctonia solani* growing in the pore space of a sandy loam. Scale bar 20 μm (Harris et al., 2002. Reproduced with permission of the British Mycological Society). **(B)** Micrograph of *ethidium* bromide-stained thin sections of a silt loam soil after inoculation by *Escherichia coli*. Image obtained using an epifluorescence microscope with blue excitation (Modified from Li et al., 2004. Reprinted with permission). **(C)** CARD-FISH stained *Bacillus subtilis* cells in soil filter sections under double excitation filter 643 (465–505 and 564–892 nm) (Modified from Juyal et al., 2018).

Given the technical difficulties associated with 3D measurements, it is natural that researchers attempted to find out what information could be obtained from 2D cuts through soils. Alexander and Jackson (1954, 1955) were apparently the first to suggest that thin sections of resin impregnated soil samples could be useful to observe algae, fungal hyphae, and bacteria, using either light or phase-contrast microscopy. They indicated that staining the soil before impregnation enhances the detection of hyaline mycelia and bacteria. Nevertheless, as soon as transmission and scanning electron microscope became available, microbiologists turned to the machines to obtain information about soil microorganisms. Consistently, they confirmed Clark’s (1951) observations. Bacteria, often coated by clay platelets, were generally present, not as “biofilms,” but as small colonies of a few cells, with many bacterial cells being dispersed in the rest of the soil as individual cells (Foster, 1988). As enlightening as these and other similar observations have been and still are^5^, a drawback with TEM and SEM is the fact that at least until recently they could provide only *qualitative* information. To obtain *quantitative* data about the distribution of microorganisms, researchers found it necessary to return to staining cells in thin sections that could be georeferenced easily and viewed in their entirety (e.g., Jones and Griffiths, 1964; White et al., 1994; Nunan et al., 2001, 2002, 2003; Li et al., 2003, 2004). Aside from non-specific stains like calcofluor white M2R applied before impregnation (Postma and Altemuller, 1990), or basic fuchsin and methylene blue applied after impregnation (Tippkötter et al., 1986), researchers also have been interested in selective staining techniques of specific cells, e.g., using fluorescence-conjugated antibody techniques (Postma and Altemuller, 1990), to observe the distribution of bacteria and fungal hyphae in soils. In some cases, problem arose because of the crystallization of the stains when in contact with soils (Harris et al., 2002, 2003). Nevertheless, after these slight technical issues got resolved, images of soil thin sections obtained with these various staining techniques showed clearly that for fungal hyphae, given their size, it is relatively straightforward to identify them (**Figure 8A**). But for bacteria and archaea, as clearly indicated in **Figure 8B**, a tremendous amount of skill (or faith, or both) is required to be able to identify a cell conclusively. Experience shows that part of the problem is related to the difficulty, with traditional light microscopes to focus precisely on a specific depth. It is possible, but tricky, to focus on the top surface of a thin section, hoping that one would then have a sharp image of the first 1 or 2 μm at the surface (Nunan et al., 2001). An easier solution consists of using a confocal laser microscope (e.g., Caldwell et al., 1992; DeLeo et al., 1997; Li et al., 2004), which can produce sharp 2D images at selected shallow depths within a soil thin section. With special software, 3D images can be reconstructed from a set of z-dependent 2D images. In principle, an extension of Hapca et al.’s (2015) statistical interpolation technique, described earlier, should make it possible to assemble these very thin 3D images into a full 3D picture of microbial microscale distribution in soil columns.

A more difficult problem to resolve with traditional stains is related to their non-specific binding to OM or other soil constituents. As a result, many microbial cells may be undetectable against a very bright background of fluorescing soil constituents (**Figure 8B**). Luckily, that stumbling block too has found a solution in recent years, with the development of very reliable fluorescence *in situ* hybridization (FISH) techniques, which use fluorescent-labeled oligonucleotide probes (Pickup, 1995; Mcnaughton et al., 1996; Li et al., 2004; Eickhorst and Tippkötter, 2008a). Eickhorst and Tippkötter (2008b) have shown that when FISH is combined with tyramide signal amplification, in what is referred to as Catalyzed reporter deposition (CARD)-FISH, one typically obtains higher signal intensity and reduced interference of the background fluorescence of the soil. This methodology, or variants of it, have been used by a few researchers in the past decade to assess the distribution of bacteria in soils (Schmidt et al., 2012a,b, 2015; Schmidt and Eickhorst, 2014). The combination of CARD-FISH, or one of its variants, with confocal laser microscopy, affords a very powerful tool to visualize and quantify the distribution of microorganisms in soils, with a satisfactory depth-resolution.

The (few) measurements that have been carried out in thin sections have shed some light on the spatial pattern of microbial distribution in soils, but many questions remain. Based on the analysis of 744 images of observed bacterial distributions in soil thin sections taken at different depths, Raynaud and Nunan (2014) found that the distance between neighboring bacterial cells was, on average 12.46 μm and that these inter-cell distances were shorter near the soil surface (10.38 μm) than at depth (>18 μm), due to changes in cell densities. These authors’ analyses suggest that despite the very high number of cells and species in soil, bacteria only interact with a few other individuals. For example, at bacterial densities commonly found in bulk soil (10^8^ cells per gram of soil), the number of neighbors a single bacterium has within an interaction distance of ca. 20 μm is relatively limited (120 cells on average). This analysis is based on calculations of Euclidean distances, which as Raynaud and Nunan (2014) acknowledge do not take into account the presence of solids, nor the tortuosity of the pore space.

A slightly different perspective on the distribution of bacteria is obtained when one proceeds to a simple back-of the-envelope calculation focused on the surfaces of soil pores, which may indicate somewhat better than Euclidean distances the degree of separation among bacterial cells. The specific surface area of soils varies between a low of 0.1 m^2^ per gram, for coarse sand, to a high of 800 m^2^ for a smectite clay, with most soils falling in between these extremes (Pennell, 2016). In a soil with a relatively low specific surface area of 10 m^2^ per gram, a population of 10^8^ bacteria, each having on average a 1 μm^2^ cross section, would occupy a mere 0.0001 m^2^ per gram of soil, i.e., about 1/100,000th of the specific surface area. Even if one assumes bacterial cells to be much bigger, with a longitudinal cross-sectional area of 4 μm^2^, they would still cover only 1/25,000th of the specific surface. In other words, in either case, it is as if on the surface of soil solids, each cell would sit in the middle of an exclusion zone with an average radius of 178 μm. These numbers, in line with earlier estimates (Postma and van Veen, 1990; Grundmann, 2004; Young and Crawford, 2004; O’Donnell et al., 2007; Vos et al., 2013; Kuzyakov and Blagodatskaya, 2015) suggest a very lonely existence indeed, but of course, they are very crude estimates at best, ignoring any tendency cells may have to aggregate. Nevertheless, as more micrographs like that of **Figure 8C** become available in the coming years, and are combined with detailed information about the geometry of the pore space, it will become possible to refine our understanding of the patterns of spatial distribution not only of bacterial cells, but also of archaea, fungi, and bacteriophages.

### Background: Dominant Paradigm and Slow Shift to a New One

The relatively low number of articles dealing with the distribution of microorganisms in soils may surprise, especially given the tools that have been at our disposal for at least a decade (CARD-FISH) or even two (confocal laser microscopes). To understand why that has been the case, and especially to try to change this unfortunate state of affairs, it is useful to describe briefly in what context this work has been carried out. This background is of course familiar to soil microbiologists, but researchers in other disciplines may not necessarily be aware of it.

In the mid-1960s, contrary to the views that had prevailed earlier, a number of researchers, seeking to make their life easier, decided to start viewing the soil microbial biomass as a blackbox, which meant ignoring entirely both the diversity of microorganisms present in soils and their relation to their immediate physico-chemical environment (Baveye, 2018). This approach became dominant for a few years, but in the 1980s and 1990s, a slew of molecular methods were developed to characterize DNA or RNA extracted from soils (Maron et al., 2011; Mendes et al., 2015). Microbiologists in growing numbers jumped on these methods enthusiastically, with the hope that they would give them the opportunity to get information about the diversity of soil microorganisms, i.e., would allow them access inside the blackbox of soil biomass, but still with the convenience of not having to worry about where exactly microorganisms are located. Indeed, in what became known as “metagenomic” and, more recently, “high throughput sequencing” methods, all that was needed to carry out the analysis of a given soil was to extract its microbial DNA or RNA. Indeed, virtually all researchers adopting this approach have entirely ignored the geometry of the pore space in soils or the characteristics of microenvironments (e.g., Nannipieri et al., 2003). Even the so-called “high-resolution” metagenomics (Kalyuzhnaya et al., 2008) ignores the physico-chemical environment of microorganisms. The claim was also made initially, at least by some, that metagenomic methods would enable researchers to avoid having to culture microorganisms in the laboratory, a process that for an estimated 98% of soil microbes, had proven impossible until then (Vogel et al., 2009).

In terms of actual benefits of work carried out along those lines, one should mention the fact that knowledge of the diversity of nucleic acids present in soils paved the way for the design of oligonucleotide probes used in FISH. In itself, this is an important outcome, but in most other ways, experience over the years has demonstrated that many if not all of the initial claims made by proponents of metagenomics were unrealistically optimistic. Scores of researchers have shown that the extraction of DNA or RNA from soils in many cases manages to get at only a fraction of the total amount present (Terrat et al., 2012; Knauth et al., 2013; Dlott et al., 2015; Wagner et al., 2015), that some of this DNA or RNA material is associated with dead or dormant cells, or is extracellular (Carini et al., 2016), and finally, that the information yielded by DNA or RNA analysis provides a picture of the genetic potential of microorganisms in a soil, not at all of what microorganisms actually do (Prosser et al., 2007; Blazewicz et al., 2013). Furthermore, it has become obvious that, far from alleviating the need to culture microorganisms, the metagenomic approach has increased the urgency of finding ways to identify and characterize vastly more organisms than is the case at the moment (Oremland et al., 2005; Baveye, 2009a,b; Pham and Kim, 2012; Puspita et al., 2012; Prakash et al., 2013). In spite of all these false hopes, limitations, and biases (Lombard et al., 2011; Prosser, 2015), it is fair to say that, at the moment, bulk “meta”-something-“omics” approaches (metagenomics, metatranscriptomics, metaproteomics, metabolomics) capture virtually all the funding dedicated to soil microbial diversity, and their use seems to be crucial to insure microbiologists’ career advancement. Year after year, articles praising the merits of metagenomics to uncover the secrets of soils (e.g., van Elsas et al., 2008; Vogel et al., 2009; Delmont et al., 2011; Fierer, 2017; Popescu and Cao, 2018) still make headlines^6^ and attract record numbers of citations.

But things may be changing. Roughly 12 years ago, partly in response to the inability of metagenomics to link functions to species and also because information needed to make sense of metagenomics data is lacking for a multitude of still uncultured microorganisms (Su et al., 2012; Shi et al., 2015), researchers started investigating ways to isolate and sequence the DNA and RNAs of individual cells. A number of articles (e.g., Wang and Bodovitz, 2010; Lasken, 2012, 2013; Pamp et al., 2012; Stepanauskas, 2012, 2015; Yilmaz and Singh, 2012; Blainey, 2013; Woyke et al., 2017) have recently retraced some of the key breakthroughs that have enabled what could probably be viewed as a fundamental revolution, in particular in the application of molecular biology techniques to environmental systems (Ishoey et al., 2008). The onset of that revolution is generally considered to be Raghunathan et al.’s (2005) proof-of-principle demonstration that it is possible to use the multiple displacement amplification (MDA) reaction to amplify genomic DNA from a single bacterium several billion fold, with a recovery of about 30% of the genome in the process. Marcy et al. (2007), Podar et al. (2007), and Kvist et al. (2007) applied MDA to environmental cells and established the feasibility of single-cell genome sequencing from uncultivated targets. Woyke et al. (2010) showed that it is possible to produce a completely closed genome from an individual cell. Progress has been very rapid since (**Supplementary Figure S1**), including in RNA sequencing (e.g., Pan et al., 2013; Svensson et al., 2017), single-cell transcriptomics (e.g., Kang et al., 2011), and single-cell metabolomics (e.g., Heinemann and Zenobi, 2011).

All these single-cell techniques offer tremendous potential for the study of soil microorganisms as various researchers have already pointed out (e.g., Ishii et al., 2010; Pedersen et al., 2015), provided two key challenges can be overcome. The first is related to the fact that FISH, the technique that seems most promising at the moment to locate bacteria and archaea in 2D cuts through soils, has been documented to interfere with single-cell genome recovery (Woyke et al., 2017). This problem may be partly avoided by complementing FISH with other methods to detect and characterize microorganisms, like Raman spectroscopy (see below). The second challenge is related to isolating individual cells from their microenvironments in soils. In the past, various researchers have used micromanipulators of different types over the years, to extract hyphae fragments from soils (Söderström and Erland, 1986) or to sample bacteria on soil surfaces (Dennis et al., 2008). Ashida et al. (2010) and Nishizawa et al. (2012) adopted a micromanipulator originally developed by Fröhlich and König (1999, 2000) and Ishøy et al. (2006), and consisting of a microcapillary (with an outside tip diameter of 60–100 μm), to extract individual, artificially elongated bacteria from a rice paddy soil sample, and subsequently proceed to 16S rRNA gene analysis. More recently, Ringeisen et al. (2015) have used a laser printing technique, called BioLP, to isolate viable microorganisms from a thin layer of soil spread over a titanium-coated quartz plate. For both the micromanipulator and laser printing technologies, the technological challenge at this point is to design a sampling method that would have a far smaller footprint than is currently achievable, to make it possible to zero in on a single cell or a very small group of cells in a soil microhabitat. Since intracellular capillary microsensors with tip diameters less than 1 μm have been used by microbiologists for at least 60 years (Draper and Weidmann, 1951) and the technology of “optical tweezers” has evolved tremendously in the last 2 decades (e.g., Fröhlich and König, 2006; Whitley et al., 2017), it may not be foolish to imagine that we could come up with a way to extract single bacterial cells from soils in a very efficient manner in the not too distant future.

An argument that could be put forth to downplay the interest of this type of single-cell analysis is that such a detailed description of microbial communities is an unnecessary luxury for understanding a large number of microbial functions in soil. It is widely accepted that microbial communities are characterized by a functional redundancy with respect to a range of functions, such as organic C mineralization (e.g., Wertz et al., 2006, 2007; Allison and Martiny, 2008), meaning that the loss of a large number of species does not have a significant effect on functions of interest. Where functional redundancy is apparent, it is possible that viewing microbial communities in soil as a distribution of active sites rather than a distribution of species might suffice. Further research is needed to determine under what conditions information about individual microbial cells is crucial and when it is superfluous.

### Dynamical Picture: Are Micromodels a Way Forward?

Given the need to impregnate soils with resin and to cut through the resulting block in one way or another to obtain information about the distribution of microorganisms and the (bio)chemical features of the microenvironments where they reside, it is clear that it is not possible at this stage to monitor in real time, at the microscale, the dynamics of microbial processes in actual soils. And, to be realistic in our expectations, for bacteria, archaea, and definitely viruses, it may be that we shall never be able to monitor their activity directly in soils.

We might be able, however, to observe the dynamics of these organisms or viruses in 2-dimensional, manufactured soil-like structures, generally referred to as micromodels or “microfluidic” devices. The development of these micromodels has been the object of significant research over the last 20 years (Karadimitriou and Hassanizadeh, 2012; Stanley et al., 2014, 2016; Stanley and van der Heijden, 2017; Aleklett et al., 2018). Early generations of micromodels, still in use to some extent (e.g., Dupin and McCarty, 1999; Stewart and Fogler, 2001; Lanning and Ford, 2002; Coyte et al., 2017; Borer et al., 2018) had idealized geometric properties, being basically two-dimensional networks of straight cylindrical segments, etched in glass or plexiglass. But as technology matured, second- and third-generation structures have become progressively closer to what one would find in a typical fine- to medium sandy soil. Of course, the material these micromodels are made of, often polydimethylsiloxane (PDMS), does not have the same surface properties as sand or silt particles. For some microbial processes this may be an issue, and it makes it impossible to reproduce surface chemical properties of soils, but at least the geometry of the pore space is realistic. With such a soil-like micromodel, Deng et al. (2015) have been able to observe the effect of extracellular polymeric substances (EPS), released by bacteria, on the drying kinetics of the pore space, whereas Rubinstein et al. (2015) have used it to demonstrate the effect a protist, the ciliate *Colpoda* sp., can have on the transport of nanoparticles through soils.

The use of micromodels opens up a number of very interesting avenues for further research, which may provide useful insight. For example, micromodels would seem to be ideal systems to instrument with optodes (Pedersen et al., 2015; Rubol et al., 2016), in order to access some of the physico-chemical parameters (pH, redox potential) that at the moment we cannot measure in soils at the microscale. The use of micromodels might also allow us to better understand how the moisture content of soils influences the activity of bacteria, archaea, and fungi. There is macroscopic evidence that these organisms react very differently to high or low moisture contents (e.g., Otten et al., 1999; Otten and Gilligan, 2006; Kaisermann et al., 2015; Baveye et al., 2016b), as do their predators, which we should not forget (Stefana et al., 2014), and it would be very useful to obtain direct evidence of this at the pore scale.

However, it is likely that for micromodels to give us valuable insight about actual soils, at least two significant challenges will have to be addressed and resolved. The first concerns the connectivity of the pore space. In nature, microorganisms evolve in a 3-dimensional space, which is significantly more connected than is achievable in 2D (e.g., discussion in Hapca et al., 2011). It will therefore be crucial to find a way to relate 2D observation made in micromodels with the more complex situation found in soils. The second challenge is related to what was referred to as “sub-resolution” pores in CT images. The issue, still very much an object of debate, is whether these pores are important to understand microbial activities in soils, and therefore whether they should be present in micromodels. A body of literature, published over the last few decades, argues that pores in the 30 to 150 μm size range are particularly crucial to understand microbial activity (e.g., Kravchenko and Guber, 2017). Specifically, pores of this size group were found to harbor greater abundance of a number of bacteria groups, such as copiotrophic actinobacteria, firmicutes, and proteobacteria (Kravchenko et al., 2014b) and presence of such pores was associated with greater microbial activity and greater OM decomposition (Killham et al., 1993; Chenu et al., 2001; Strong et al., 2004; Ruamps et al., 2011, 2013), in spite of a higher predation pressure in larger pores, compared to small ones (e.g., Wright et al., 1995). Recently, it was also found that dissolved OM contained within such pores is more labile, having less lignin and tannin-like compounds, than that in small (<6 μm) pores (Bailey et al., 2017; Smith et al., 2017). One perspective on these data is that pores of this size range offer better micro-environmental conditions, e.g., O_2_ and water supply, while providing enough space not only for individual organisms but for formation of microbial colonies, which then generate these sizeable experimentally detectable activities and changes in soil characteristics.

Based on this evidence, one would be temped to conclude that when constructing micromodels, one could safely ignore small pores, which would undoubtedly make everyone’s life simpler. However, the much lower connectivity of the pore space that would result from that may prevent us from describing correctly some of the processes occurring in soils, both in terms of microbial movement and metabolism. To resist predation, it may be vital for bacteria and archaea to be able to find refuge in smaller pores in which amoebae and particularly ciliates are not able to penetrate. One expects motile bacterial and archaeal cells, sometimes as small as 0.3 μm in diameter or width in soils, to be able to move relatively easily in and out of 2–3 μm-wide pores filled with water. But, as the experiments of Männik et al. (2009) with micro-fabricated channels show, some bacterial cells (of *Escherichia coli*, but not of *Bacillus subtilis*), can penetrate pores smaller than themselves. Although organisms constricted in narrow channels had no mobility and were squeezed, they could still penetrate the channel by growth and division (Hallett et al., 2013). Perhaps more important still is the fact that, given their even smaller size, exoenzymes that bacteria and archaea, as well as fungal hyphae, release into the soil solution can move in and out of tiny pores. Likewise, solutes present in the soil solution can diffuse in and out of the smaller pores, including the very narrow 1.8 nm-wide spaces between clay particles (Dumestre et al., 2000, 2006). In particular, dissolved components of the OM that is located, and possibly to some extent is physically protected, in small pores can also diffuse out into wider pores, where they can be taken up by microorganisms or be transported with the percolating water. Results obtained by Michelson et al. (2017) using a microfluidic device also suggest that members of the *Geobacteraceae* family produce nanowires that are able to penetrate in pore spaces too small for cell passage and, there, up to 15 μm away from cell bodies, reduce Mn(IV) and Fe(III) oxides via long-range extracellular electron transport. Finally, the (so far virtually ignored) bacteriophages swarming in the pore space of soils in huge numbers (Ashelford et al., 2003), with sizes sometimes as small as a few tens of nanometers, are likely to diffuse through tiny pores as well, and it may turn out that to understand the dynamics of phages in soils, a topic of increasing interest at the moment, it will be necessary to deal with sub-resolution pores in one way or another.

For all these reasons, inclusion of sub-micron pores in micromodels is a technological challenge that may need to be met if we want to use micromodels to gain knowledge about a range of soil processes, but in the meantime, a number of interesting processes, which are not or are only marginally influenced by sub-resolution pores, can still be studied with existing soil-like micromodels, such as the proliferation of fungal hyphae (as long as the release of exoenzymes is not the key mechanism by which fungi metabolize food sources), or the effect of bacterial activity on water or particle retention and movement in larger pores (e.g., Deng et al., 2015; Rubinstein et al., 2015).

### Modeling of Microbial Spread and Activity in Soil Pores

Contrary to what has happened on the (bio)chemical scene, the least one can say is that the lack of experimental data about the spatial distribution and activity of microorganisms in soils has not discouraged at all a number of researchers from developing increasingly more sophisticated biokinetic models. On the contrary, work in this area, overwhelmingly carried out by soil physicists, mathematicians, and computer scientists, has been extensive over the last decade (e.g., Thullner and Baveye, 2008; Heße et al., 2009; Gras et al., 2010, 2011; Wang and Or, 2010; Gharasoo et al., 2012; Ebrahimi and Or, 2014, 2015, 2016, 2017; Vogel et al., 2015; Tecon and Or, 2017a,b; Wilmoth et al., 2018; Vogel H.J. et al., 2018; Vogel L.E. et al., 2018). Some of this research has consisted at first of a relatively straightforward extension to the microscale of macroscopic modeling approaches originally developed for saturated porous media, with the biomass consisting exclusively of bacteria, attached to surfaces and growing in response to the influx of a substrate, according to Monod’s equation in its simplest formulation (e.g., Widdowson et al., 1988; Baveye and Valocchi, 1989; Loehle and Johnson, 1994; Vandevivere et al., 1995). Over the years, in addition to being extended to the microscale in variably saturated porous media, the original biokinetic model has also been greatly improved (e.g., Hron et al., 2015). Description of bacterial growth has included an explicit account of endogenous metabolism. Bacteria have been allowed to move via chemotaxis, in response to substrate concentration gradients (Olson et al., 2004; Ebrahimi and Or, 2014; Son et al., 2015), and to become dormant (Gras et al., 2011; Resat et al., 2012; Joergensen and Wichern, 2018), under a range of conditions. Instead of simply relying on population-level kinetic equations like Monod’s, researchers have progressively turned to individual- or agent-based models, recognizing that locally in soils, the number of bacterial cells tends to be very small, and therefore the large-number assumption embodied in Monod’s equation is no longer met (Hellweger et al., 2016). In all these respects, progress in the development of the models over the last decade has been very significant, although from a strictly bacteriological perspective, the models currently available still fail to include a number of processes that might be very significant in soils, like conjugation, quorum sensing, siderophore production, exopolymer and exoenzyme production, filamentous growth of some bacterial strains, or the release of antibiotics to compete with other bacteria or archea (Wolf et al., 2013; Abrudan et al., 2015; DeAngelis, 2016).

In parallel with this modeling effort related to bacteria, various researchers have endeavored to develop computer models to describe the 3-dimensional proliferation of fungi in various types of environments (Otten et al., 2001; Falconer et al., 2005, 2007, 2012, 2015; Boswell and Hopkins, 2008; Jeger et al., 2008; Pajor et al., 2010; Kravchenko A. et al., 2011; Kravchenko A.N. et al., 2011; Hopkins and Boswell, 2012; Cazelles et al., 2013; de Ulzurrun et al., 2017). These models include a number of processes, which for soils might be very relevant, like biomass recycling and the release of exo-enzymes. In applications of some of these models to soils, thresholded CT images can be used to establish the boundaries of the geometric domain in which fungal growth occurs.

Since most soils simultaneously harbor bacteria, archaea, and fungi (among many other organisms), one would expect that the two families of models developed so far to describe specifically the activity of these organisms would have been combined at some stage. This would seem to make a lot of sense, especially as far as bacteria are concerned. One might argue that, under a number of circumstances (e.g., discrete POM serving as exclusive carbon source to fungi), the presence or not of bacteria in the pore space is in general pretty much irrelevant for the proliferation of fungal hyphae. Exceptions occur when bacteria have fungicidal activity (Stanley et al., 2014), and influence the propagation of hyphae. But it is more common for fungi to exert an influence on the behavior and spread of bacteria. Over the last few years, evidence has accumulated that bacteria, “Hitchhikers on the fungal highway” as Warmink et al. (2011) put it, can hop on, or at least be passively carried by, fungal hyphae as they propagate through the pore space (Kohlmeier et al., 2005; Warmink et al., 2011; Ellegaard-Jensen et al., 2014; Stanley et al., 2014), with the consequence that mycelia may be having a very significant role in gene transfer in soils (Berthold et al., 2016; Nazir et al., 2017). Recent ToF- and NanoSIMS measurements carried out by Worrich et al. (2017) also demonstrate that fungal or fungal-like (oomycete) mycelia can reduce water and nutrient stresses experienced by bacteria in otherwise dry and nutrient-poor microhabitats. All these recent observations seem to run counter to previous research suggesting that the high biodiversity of bacterial populations in soils, as well as their community structure, could be accounted for by the low connectivity of the water-filled pore space (e.g., Tiedje et al., 2001; Fierer et al., 2003; Treves et al., 2003; Carson et al., 2010; Ebrahimi and Or, 2015).

Therefore, it would seem important for models describing the activity of microorganisms in soils to simultaneously involve bacteria, archaea, and fungi, and in particular describe the transport of bacterial or archaeal cells by fungal hyphae or the transfer of water and nutrients by mycelia in the pore space. Unfortunately, this is far easier said than done, because of the difference in scale at which these various groups of organisms operate. In order to describe the activity of fungi realistically, one needs to model a volume of soil in which typically very large numbers of bacterial or archaeal cells would be located, making the prediction of microbial activity extremely CPU intensive, especially when using individual-based models. Within the context of this type of model, there is a possibility to deal with groups of cells, or “super-individuals,” as if they were single individuals, to save computing time (e.g., Scheffer et al., 1995) but the approach does not appear to have been used yet to describe bacterial or archeal populations in soils, and it remains to be seen whether it really makes sense.

This hurdle we need to resolve, somehow, about including both bacteria and fungi in the same simulations, raises a broader question of how many other similar hurdles we need to face. How much biodiversity needs to be included in models, in order to have meaningful insights into what is occurring in soils, and in order for the label of “microbial” used abusively in the title of many articles dealing only with bacteria (e.g., Or, 2002; Ebrahimi and Or, 2015), is really justified? In principle, there is no problem in developing models that involve only one type of microorganism, as long as the conclusions reached are restricted to the organism(s) involved, under the conditions assumed in the modeling, and are not considered generally applicable to soils, which contain a multitude of other organisms beside the targeted one(s) (this point is discussed in detail in Baveye et al., 2016b). Clearly, however, such limited models, from which crucial components are missing, are not likely at all to be very useful in the long run in the context of the program defined in **Figure 1**. To make real progress, we need a model that includes as many as possible of the organisms that are relevant to the goal that is being pursued. In general, bacteria, archaea, fungi probably all need to be included, but so do their predators (e.g., DeLeo and Baveye, 1997; Ronn et al., 2012), as well as bacterial and archaeal phages that are present in the soil in large numbers and are more and more suspected to have a very significant, yet still largely misunderstood, influence on microbial dynamics (Williamson et al., 2017; Pratama and van Elsas, 2018). Likewise, the too often ignored aspects of mesofaunal and macrofaunal activity in soils (Briones, 2014), which directly relate to the growth and metabolism of microorganisms, probably also ought to be accounted for, somehow. Depending on the specific questions we try to address, it may be that, in addition to microorganisms, phages and the mesofauna all need to be taken into account in our description of soils, in which case individual-based techniques might not be workable, or only some organisms need to be involved explicitly. Further research is needed to enlighten us in this respect.

As we navigate among all these additional components that may, or may not, need to be added to current microbial models to make them encompass more of the known biodiversity of soils, it soon becomes apparent that progress vitally requires being able to compare model predictions with actual measurements, which at this point, as was discussed in previous sections, are sadly lacking… Over the years, various soil scientists have reacted strongly, sometimes eloquently (Thomas, 1992), sometimes caustically (Philip, 1991), against modeling efforts that are not systematically backed by sound experimental support. A well-known philosopher and writer, David Henry Thoreau, offered a long time ago a more positive take on a similar situation (in a different context), when he wrote: “If you have built castles in the air, your work need not be lost; that is where they should be. Now put the foundations under them.” (Thoreau, 1854). Clearly, in the case of models of microbial activity in the hugely complicated environment that soils constitute for microorganisms, it seems essential to heed this advice, and to obtain relatively quickly the type of experimental data that would enable us to establish our modeling efforts on a much stronger foundation.

### Visual Summary of the Status of the Microbiological Front

In terms of measurements, the situation on the microbiological front is very similar to that found on the (bio)chemical one (**Figure 5**). Quantitative measurements of microbial distribution or dynamics are extremely limited and related only to a very small portion of the biodiversity found in soils. Unlike on the (bio)chemical front, however, efforts to model the activity of microorganisms in soils have been extensive, especially regarding bacteria, and have produced some interesting predictions. Nevertheless, this effort has so far been entirely focused on selected bacteria and just a few species of non-sporulating fungi, and in the absence of actual measurements, it is not clear at all how close to reality model predictions are.

## Integration and Modeling of Multiple Scenarios

The next step in the program of **Figure 1** consists of integrating disciplinary insights into a coherent integrated picture of microbial processes in soils. This integration should take place at both the static and dynamic experimental levels, and in terms of modeling, with the understanding that what is needed eventually, going into the next step, is a comprehensive, thoroughly tested microscale *model* of microbial activity. Right from the onset, one should expect this integration to pose significant challenges. Besides the usual institutional impediments to any kind of interdisciplinary research (see, e.g., Baveye, 2013b, 2014; Baveye et al., 2014), this integration is complicated by the fact that separate measurements that need to be made on the same soil samples often require heavy pieces of equipment that are not commonly found in a single location, causing logistic issues.

At the experimental level, one would expect that since data are still scanty on the (bio)chemical and microbiological scenes, very little integration would have taken place. And yet, encouragingly, some countries have set up a framework for efforts along these lines (e.g., Kögel-Knabner et al., 2008) and there have already been several attempts at integrating various types of experimental approaches. A case in point is the very interesting article by Rawlins et al. (2016). These authors attempt to determine how soil heterotrophic respiration (SHR) is related to the accessibility of OM to microbes in aggregates of a soil from the United Kingdom. They use a combination of synchrotron X-ray CT, osmium staining, and total organic carbon (TOC) content measurements to quantify the 3D distribution of OM, pore space, and mineral phases, and eventually find a weak correlation (*r* = 0,12) between SHR and a measure of accessibility of OM, which they define as the probability that a given voxel, “filled” with OM be adjacent to a pore voxel. More recently, Yu et al. (2017) combine synchrotron-based 3D X-ray micro-computed tomography with scanning electron microscopy of 2D slices of two different soils, coupled with an energy –dispersive X-ray spectrometer (SEM-EDX) to establish the relation between pore architecture and cementing substances (iron oxide, carbon) in soil aggregates. In an even more recent article, Vidal et al. (2018) combine Nano-SIMS to FIB-SEM to gain information about the distribution of minerals and biomass in the vicinity of roots. They show in particular that bacteria near roots are surrounded by iron oxides, and that some microaggregates are intimately associated with the surface of fungal hyphae.

In terms of the integration of models, some limited work has been carried out as well. Falconer et al. (2012) focus on combining predictions of water retention in a soil, using a LB approach, with a model of the growth of fungal hyphae. Simulation results, based on X-ray CT images of three different soils, show that the water distribution in the soils is affected more by the pore size distribution than by the total porosity of the soils. The presence of water decreases the colonization efficiency of the fungi, as evinced by a decline in the magnitude of all fungal biomass functional measures, in all three samples. The architecture of the soils and water distribution have an effect on the general morphology of the hyphal network, with a “looped” configuration in one soil, due to growing around water droplets. These morphologic differences are satisfactorily discriminated by Minkowski functionals, applied to the fungal biomass.

**FIGURE 9:**
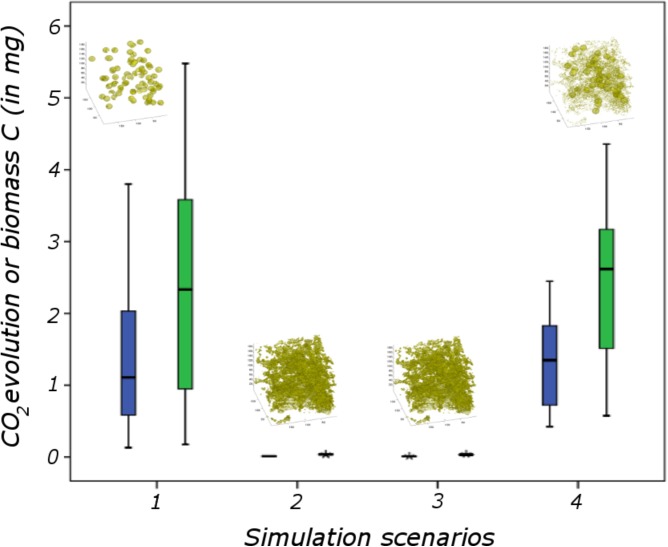
Boxplot diagram of simulated CO_2_ production by fungi in soil samples with an identical Particulate Organic Matter (POM) content of 3%. The abscissa represents different scenarios with, respectively, (from left to right) 49.95, 51.06, 56.73, and 60.12% of POM accessible at pore-solid interfaces. Individual samples differ in the way POM is distributed in the pore space (Modified from Falconer et al., 2015).

Two other articles, also combining different models, demonstrate the large benefits that can be derived from the availability of models. Once an X-ray CT image of a soil have been obtained, one can artificially create all kinds of “what-if” scenarios, in which one can imagine that the OM or the microorganisms are distributed in the soil in a multitude of different manners, and one can determine the effect that these relative distributions have on some macroscopic outcome, like the amount of CO_2_ evolved from a given soil sample. Of course, such “what-if” scenarios do not alleviate the need to secure actual measurements, of microbial and OM distribution, as well as of any macroscopic outcome one is interested in, but the scenarios can definitely complement and expand the experimental data set in very advantageous ways, if only for the purposes of testing statistically various types of novel metrics of microscale heterogeneity (discussed later on). For example, Falconer et al. (2015) obtain strikingly different predictions of evolved CO_2_ and fungal biomass production in soils, depending on how an identical amount of POM is distributed spatially in the pore space (**Figure 9**). When POM is present in relatively large chunks (>200 μm in diameter, in scenarios 1 and 4), results in terms of both CO_2_ evolution and biomass C produced show a large variability and in some cases a high level of production. On the contrary, when an identical amount of POM is more finely dispersed in the soil sample (scenarios 2 and 3), CO_2_ evolution and biomass C production both vanish. This result may surprise, but as discussed by Falconer et al. (2015), it is entirely consistent with the foraging pattern of fungi. In another article also published just a few years ago, Vogel et al. (2015) analyze numerically the role of meso- and macropore topology on the biodegradation of a soluble carbon substrate in variably saturated and pure diffusion conditions. The simulations involve the coupling of a LB model to describe the retention of water in the pore space, and a simplified compartmental biodegradation model that does not allow bacterial motility. Not unexpectedly, Vogel et al. (2015) show that under these conditions, the biodegradation of the solute is strongly dependent on the separation distance between bacteria and solute, and is influenced by the moisture content of the soil.

### Visual Summary of Integration

To summarize this inevitably quick overview of the work done so far on the integration of disciplinary perspectives on soils at the microscale (**Figure 5**), it seems fair to say that very little progress has been achieved, in large part because very few research projects have so far focused on integration. Such an integration is already largely feasible, for example by measuring in the same soil samples, both the characteristics of the pore space and the spatial heterogeneity of physico-chemical parameters, or either one of these parameters and the spatial distribution of microorganisms. And yet, at the time of the writing of this review article, very little integration at all has occurred at the experimental level. The tiny bit of work that has been carried out is encouraging, but a whole lot of research remains to be done. This is even more so in the case of upscaling, which remains a virtual *terra incognita*.

## Upscaling, How?

### Representativeness of Observations and the Imperative of Upscaling

To achieve a high resolution when scanning soil samples, it is necessary to restrict the size of the samples. Typically, with table-top scanners, samples cannot have a volume larger than 100 cm^3^ to obtain micrometer resolution, and they have to be significantly less than that if one wants to reach the highest resolution (of about 0.3 μm) that is advertised by manufacturers. Meanwhile, the modeling of soil samples with lattice Boltzmann models is often limited at the moment to handling images of at most 500 × 500 × 500 voxels, which at a resolution of say, 20 μm, corresponds to a physical volume of only 1 cm^3^.

This small size of soil samples has occasionally raised questions in terms of the “representativeness” of measurements or simulations carried out with these samples (e.g., Al-Raoush and Willson, 2005; Costanza-Robinson et al., 2011; Gao et al., 2014; Rab et al., 2014). These questions stem from the concept of Representative Elementary Volume (REV), which has served over the last few decades as a conceptual foundation for much of the description of transport processes in soils (Fowler, 1997; Vogel and Ippisch, 2008; Hemes et al., 2015; Tracy et al., 2015; Daly et al., 2016; Liu et al., 2016; Cooper et al., 2017; Gonzalez et al., 2018). From that standpoint, soil samples scanned via X-ray CT or simulated via LB, which are of a much smaller size than the REV, would not be sufficiently representative, and one should therefore try to work with larger samples, be it physically, or virtually by aggregating together, mosaic-style, images obtained on a number of small juxtaposed samples. The difficulty with this approach is that in general one has no idea how big an REV is in any particular situation or whether an REV indeed exists (Baveye and Sposito, 1984, 1985; Vogel et al., 2002; Koestel, 2017). One can try computationally to consider sequentially larger volumes of soil in CT images to ascertain that a given property, e.g., porosity or bulk density, tends to become constant as the volume grows, as was done by various authors (Baveye et al., 2002; Vogel et al., 2002). However, there is no guarantee that the REV associated with a particular soil property applies to any other parameter of interest, so that the volume-growing procedure has to be repeated in principle for every single parameter needed to fully describe soil dynamics. Therefore, what at first appears to be a sound physically based constraint on the size of soil samples turns out often not to be operationally meaningful, and the best one can do, as suggested by Baveye and Sposito (1984), is to carefully reference any observation that is made on a soil sample to the volume and shape of this sample.

Nevertheless, regardless of how one feels concerning the need to invoke the notion of REV, it is clear that observations made on cm^3^-sized soil samples are not directly relevant to answering the questions raised by soil management, all of which relate to significantly larger spatial scales. Even the root zone of individual crop plants at maturation often encompasses several m^3^ (Baveye and Laba, 2015) and many societal concerns at the moment relate to the kilometric cells of typical climate and general circulation models used to predict global environmental change, or even to the much larger scales of watersheds and continents. Therefore, there is a definite need to upscale the observations made on small soil samples to the much larger scales at which answers are needed. In the words of Wachinger et al. (2000), “a path for translating small-scale understanding into large-scale phenomenology is required.” At the moment, no solution is available for this upscaling, which turns out to be an extremely challenging step, but different options have been suggested, some of which can be eliminated right off the bat.

### Are Increasing Sample Sizes or Volume Averaging Feasible Options?

One of the approaches that could be considered as an upscaling option consists of the process of mosaicking images obtained on small-sized soil samples, so as to obtain a virtual sample of much larger size. If this procedure results in soil samples of decimetric dimensions, one could be led to assume that the microscopic information and description relevant to the soil samples has been somehow “upscaled” to the macroscopic scale. However, even though the final sample considered may indeed be macroscopic in extent, the information one gets about it essentially remains microscopic in nature and does not necessarily provide the type of simplified description of reality that is sought in **Figure 1** and that corresponds to the notion of emergence.

Another option, which has been used by numerous authors over the last two decades, consists of averaging microscopic descriptions of porous media, over either a Representative Elementary Volume or an arbitrary volume (e.g., associated with a measuring instrument), in order to obtain macroscopic variables (Ayub and Bentsen, 1999; Lichtner and Kang, 2007a,b; Golfier et al., 2009; Valdes-Parada et al., 2009; Wood, 2009, 2010; Davit et al., 2010; Baveye, 2013a; Lugo-Mendez et al., 2015). Davit et al. (2013) have shown that in terms of outcome, this approach is equivalent to another popular upscaling method involving homogenization through multiscale asymptotics (e.g., Roose et al., 2016). In the classical literature on scales in hydrology, both methods are closely associated with what is often referred to as “coarse-graining,” in analogy to a common practice in image analysis (Kitanidis, 2015). When applied to the type of soil processes we are interested in, the upshot of volume averaging, however, one looks at it, is a massive loss of information (Baveye, 2010), which takes us several steps backward in our understanding of emerging microbial processes. Indeed, if in a given soil, we perform a simple volume averaging of the concentration of a carbon source and, separately, of the biomass density, and if in so doing we ignore all the microscopic-scale information about the relative distributions of both, we are back to a situation we used to be in, with macroscopic parameters that have no causal relationship any more, and do not allow us to describe emerging processes accurately. Even if, as envisaged, e.g., by Wood (2010) and Porta et al. (2016), one goes beyond simple volume averaging, and somehow takes into account spatial fluctuations or variance within the volume in which averages are computed, leading to non-local integrodifferential equations, the results still miss some of the key ingredients that we recognize intuitively that an upscaled description of emerging microbial processes should have, in particular a quantification of the disconnect between microorganisms and their carbon/energy sources.

### Deep Learning?

Occasionally, in discussions, the suggestion is made that the very popular “machine” or “deep” learning techniques (LeCun et al., 2015; Willcock et al., 2018) could perhaps provide a way to upscale microscale modeling of soils to the macroscopic scale (Veres et al., 2015). *Machine learning* explores the study and construction of algorithms that can learn from data and make data-driven predictions. Machine learning algorithms have started to be employed in soil science, in particular for pattern analysis and image classification to predict material classes in single channel X-ray CT images (Chauhan et al., 2016) and multi-channel nanoSIMS images (Steffens et al., 2017; Schweizer et al., 2018). *Deep learning* is a class of machine learning algorithms that use a cascade of multiple layers of non-linear processing units for feature extraction and transformation; learn in supervised and/or unsupervised (e.g., pattern analysis) manners; learn multiple levels of representations that correspond to different levels of abstraction; and use some form of gradient descent for training via back-propagation.

The application of machine or deep learning techniques to soils might consist of feeding a computer with detailed information about a multitude of scenarios, like those depicted in **Figure 1**, as well as results of simulations carried out for each scenario with the integrated model. With this supply of “big data,” deep learning algorithms would in principle search for patterns through all the simulations. Based on these patterns, it would then become feasible to predict the macroscopic behavior of a soil sample on the basis of microscopic data, without having to go through the likely time-consuming effort of re-running the integrated model. This type of outcome might conceivably be useful under specific circumstances, but it clearly does not correspond to what is expected of an upscaled model in **Figure 1**, namely the ability to predict the macroscopic behavior of soil samples *based on macroscopic data*. In other words, deep learning in itself does not automatically result in true upscaling. Nevertheless, deep learning algorithms might still be useful if somehow the patterns they identify in the data could (1) be revealed explicitly, (2) be related to specific macroscopic features of the soils, and (3) help in the development of appropriate macroscopic measurement techniques. At this point, further research is needed to determine whether any one of these different conditions can be met.

### Disconnect Is the Key, but How Do We Measure It in Practice?

The research carried out to date, and in particular some of the scenario modeling alluded to earlier, point to a “disconnect” between microorganisms and their carbon/energy sources as being one of the keys to a proper understanding of emergent microbial processes in soils. In principle, this disconnect could be quantified in a number of ways. The Euclidean distance between microorganisms and OM might be a logical candidate, but it does not suffice, since closeness does not guarantee that OM be accessible either directly to a microorganism or indirectly to its extracellular enzymes (see illustration in **Supplementary Figure S2**). To convey the degree of direct or indirect accessibility of OM to microorganisms, a possibility is to consider the length of the most direct path through the pore space that connects a given bacterial cell or segment of fungal hyphae to a blob of OM, if pore connectivity allows such a path to exist at all. This shortest path, generally referred to as the “geodesic” distance, can be computed easily for individual pairs of points, using a number of algorithms developed in graph theory. In principle, a statistical mean of all relevant geodesic distances can then be generated within a specific soil sample. Within a range of CT image resolutions (which influence the apparent connectivity of soils, and therefore the calculation of shortest paths), the mean geodesic distance may prove to have merit, in particular if its use to characterize soil samples in investigations on the effect of temperature and precipitation on carbon mineralization manages to reduce the unexplained experimental variability observed so far.

Unfortunately, the geodesic distance in itself does not provide a complete answer. One issue with it is the fact that it does not take into account the geometry of the pore space along the shortest path, with which it is associated. In practice, this geometry matters tremendously. If a given geodesic path such as the one in **Supplementary Figure S2** goes through a tiny constriction (which used to be referred to as a “pore neck”) between two adjacent voids in a soil, not only might bacterial cells or fungal hyphae have great difficulties passing through it, but chemical species (dissolved OM, exoenzymes, byproducts of enzymatic reactions) diffusing randomly through the pore space might also have a reduced likelihood of crossing over. From this perspective, instead of computing the geodesic distance, it might make more sense to quantify the average length of the path taken by molecules diffusing through the pore space. Even though conceptually, the geodesic and diffusion distances are very different, computationally they are not as distinct. Indeed, in order to compute the geodesic distance, algorithms typically track the diffusion paths of large number of random walkers, from which they eventually retain the shortest path. Therefore, the computation of an average diffusion distance between two points does not take a lot more time than the estimation of the geodesic distance.

Computer simulations, using the models under development at the moment and with a wide range of scenarios, could help determine under what conditions metrics like the mean geodesic distance, the mean diffusion distance, or some refinement of them, could be useful. The challenge at that point, then, will be to find a way to relate the metric that eventually turns out to be most suitable, to actual macroscopic measurements that make sense operationally. This is clearly a formidable challenge, whose practical importance cannot be downplayed. The end result of the program of **Figure 1** absolutely cannot require extensive microbiological, chemical, and physical measurements at the microscopic scale. To be useful, the research needs to come up with simple measurement techniques, which can be used routinely, in a fully automated mode. It is far too early to have even a vague idea of what these routine measurements might be, but they need to remain front and center on our radar screen.

## Where Are We, and What Are the Next Steps?

One way to perceive the overall message conveyed by the visual assessment of **Figure 5** is that we are not very far along the way, and that a tremendous amount of work remains to be done. One could easily argue that this “half-empty glass” perspective is more than warranted. There is indeed a lot of work left, and a long way to go. From a more optimistic, “half-full glass” viewpoint, one could contend that, given the incredible complexity of soils and the fact that suitable technologies to deal with the various components of this complexity have been available for only a little over a decade, the progress achieved to date is remarkable.

Regardless of how one feels about the current state of affairs, it seems clear what the next steps in the research should be. The first step needs to address the clear imbalance that exists among the three core disciplines in the level of effort made to secure measurements in soils at the microscale. The current uneven level of knowledge, with some aspects of the research program that are far more advanced than others, if it is not alleviated in some way, is likely to dramatically hinder the credibility of any effort to make the basic disciplinary outlooks converge into a fully integrated microscopic model. At the moment, some integration of models has taken place, but one cannot actually assess how reliable the integrated descriptions are in practice, because in most situations, relevant microbiological observations are utterly lacking. Therefore, it seems fair to say that one of the key priorities of the research in this field will be to come up with the kind of microscale observations of the distribution and activity of microorganisms that are needed, whether that work be carried out by soil microbiologists or, as it has often happened in the last few decades, by non-microbiologists who have managed to gain the required expertise. When more precise information about the location and activity of microorganisms in soils becomes available, it will be useful to try to characterize as accurately as possible the physical and (bio)chemical nature of their microenvironments, and to determine how these microenvironments co-evolve with microorganisms over time.

A second step, which should be initiated now already, without waiting for the first step to be completed, consists of running multiple analyses on the same soil samples, in order to obtain an integrated view of the different parameters that control their functioning at the microscale. Some timid efforts have been made in this respect, but we have to shift to higher speed. In most cases, given the fact that the heavy equipment (e.g., scanners, NanoSIMS) used for some of these analyses are not located in the same institutions, this integration will require soil samples and possibly also researchers to travel from one institution to another. For some time, it has become well accepted that to run synchrotron-based analysis of soils (e.g., μXRF, XANES, or NEXAFS), one had to take soil samples to one of the handful of synchrotrons around the world. But now, this same attitude will have to be generalized to a much wider range of investigations, including microbiological analyses.

The next activity we should delve into at this point, much more forcefully than has been the case so far, is to use the existing microscale models of soils to run multiple “what-if” scenarios, and thereby try to understand how, for example, a spatial disconnect between microorganisms and the POM in soils affects the mineralization of this POM. Little by little, as more and more scenarios are run, it is likely that we will progressively get a sharper idea of the features that control the emergent properties of microbial activity in heterogeneous soil microenvironments, and eventually guide us in terms of the still somewhat fuzzy (but crucial) upscaling to the macroscopic scale.

These steps should keep us busy over the next 5 years. Besides funding, several factors will determine how fast we can make progress. In particular, much could depend on how quickly we can take advantage of a number of tremendous technological advances that should become readily available to researchers in the next few years.

## Foreseeable Help From Novel Technologies?

As the preceding sections have documented in some detail, research on microscale aspects of emergent soil properties has been greatly stimulated by a number of major technological breakthroughs achieved at the turn of the century, especially in terms of X-ray CT but also with respect to other measurement techniques (e.g., CARD-FISH). The literature published in the last few years suggests that research is currently paving the way for another wave of phenomenal technological advances, which in several ways can be expected to be even more revolutionary than the previous one. Several of the new technologies are still at the development stage, such as zero-field nuclear magnetic resonance (Ledbetter and Budker, 2013) or quantum microscopes using molecular-scale MRI sensors built from diamonds (Reardon, 2017), but others have already become commercially available and could conceivably cause a huge leap in our ability to visualize and quantify processes in soils.

Years of efforts have been devoted to the development of near-synchrotron quality X-ray sources in facilities that are much smaller than the football stadium size of synchrotrons, and cost significantly less than the billions of euros a typical synchrotron does. Some of these efforts have resulted in 2015 in the installation in Munich (Germany) of the first commercially available mini particle accelerator, or “compact light source” (Eggl et al., 2016). With a very small 5 by 3 m footprint, it produces X-rays through Compton scattering, resulting from the interaction of low energy electrons and a high-powered laser pulse. The X-rays have high-brightness, intermediate between that of X-ray tube sources, used in table-top CT scanners, and large-scale synchrotrons. Another machine based on a similar principle, the ThomX compact light source is currently under completion at Orsay (France). It has an 18 m long storage ring, and will produce photons with energies up to 90 keV with a maximum flux of 10^13^ photons per second, i.e., with a brightness similar to that of synchrotrons. Undoubtedly, this type of machine will become widely available in years to come, and will eventually afford soil scientists far more access to nearly monochromatic, tunable X-ray beams than is currently the case.

X-ray beams produced using a very different approach may prove to be of even greater interest to soil scientists, because of the very small footprint and, potentially, cost, of the technology. The principle of laser-wakefield accelerators (LWFAs) was proposed more than three decades ago, and technological advances are progressively bringing them closer and closer to practical applications. In a LWFA, not much larger than a shoebox, where an intense laser pulse focused onto a plasma forms an electromagnetic wave in its wake, electrons can be trapped and are now routinely accelerated to GeV energies. Betatron motion, Compton scattering, and undulators produce tunable x-rays or gamma-rays by oscillating relativistic electrons in the wakefield behind the laser pulse, a counter-propagating laser field, or a magnetic undulator (Malka et al., 2008; Ben-Ismail et al., 2011; Mourou et al., 2013; Albert et al., 2014; Cole et al., 2015a,b; Albert and Thomas, 2016). LWFAs still need to be improved, and in particular their brightness needs to increase significantly to the level of synchrotron sources. Nevertheless, progress has been very rapid in recent years, so much so that a number of researchers have been able to use the technology for microtomography of bones, insects, and small mammals (Cole et al., 2015a,b; Wenz et al., 2015; Döpp et al., 2018). It may not be very long before LWFAs are commercialized and become viable options to image soils.

Perhaps as a result of the appearance of novel sources of X-rays, there has also been an upsurge of interest in developing a variety of novel X-ray detectors (Gruner, 2012), such as the photon-counting silicon-strip detector allowing energy-resolved CT (Persson et al., 2014). Various research groups have also been keen to look beyond simply taking advantage of X-ray attenuation to produce 3D images of materials, including soils. Phase-contrast is a very good candidate in this respect (Bhreasail et al., 2012). Techniques that have received attention recently are edge-illumination phase-contrast tomography (Zamir et al., 2017), dark-field scatter tomography (Bech et al., 2010) and ptychographic X-ray computed tomography. In the latter, phase-contrast information can be used to generate high-contrast 3D electron density maps without having to invoke the usual assumptions of a weak phase object or negligible adsorption (Chapman, 2010; Dierolf et al., 2010). To our knowledge, even though they have been mentioned in the literature on natural porous media, these techniques have not yet been applied to soil samples, but this is bound to happen in the not too distant future, at which point it will be possible to determine exactly how much promise they hold.

A possible danger with these tremendous technological advances that are now on the not-very-distant horizon is that they will allow major progress to be made chiefly in the quantification of the physical aspects of soils, thereby widening the already large gap that exists relative to the (bio)chemical and microbiological aspects. But that does not need to be the case. High-brightness, monochromatic, tunable X-ray and gamma-ray beams, possible with LWFAs, could prove extremely useful to visualize water and OM in soils. Also, it may turn out that some of the alternative X-ray techniques, like phase-contrast or dark-field imaging, may offer great advantages to visualize OM or even fungal hyphae in soils. Techniques like 3D micro-XRF or micro-XANES (Silversmit et al., 2010), whose application to characterize the (bio)chemical make-up of soils is currently handicapped by the limited access to synchrotron facilities, may also benefit greatly from the widespread availability of much cheaper, versatile X-ray sources.

Progress is being achieved not only in terms of X-ray or gamma-ray. One area where progress has been tremendous, and that, clearly, holds a lot of promise to assess the distribution of microorganisms in soil thin sections is related to fluorescence microscopy. Light microscopy, including fluorescence microscopy, has experienced phenomenal advances in the last decade. Until about 40 years ago (Cremer and Cremer, 1978), the resolution achievable with light microscopy was strictly constrained by the diffraction of light. Over time, increasing numbers of “super-resolution” microscopes have been developed, relying either on deterministic super-resolution techniques, like the stimulated emission depletion (STED) and saturated structured illumination microscopy (SSIM), or on stochastic functional techniques, like the super-resolution optical fluctuation imaging (SOFI) and the omnipresent localization microscopy (OLM) (Min et al., 2011; Cremer and Masters, 2013; Duwé and Dedecker, 2017; Ji, 2017; Power and Huisken, 2017; Yang and Yuste, 2017). Again, most of these techniques have yet to be applied to soil samples. With more and more of this super-resolution equipment becoming commercially available, there is little doubt that this application to soils will occur in the near future. When super-resolution images become available, we might be surprised (or not) to find out that the individual spots of light in images like those of **Figure 8**, which at the moment are identified as single bacterial or archaeal cells, are in fact small groups of cells.

In terms of the identification of microorganisms, significant progress has also been achieved recently, which could be very helpful in soils. In parallel with single-cell omics methods, a number of other techniques have been developed in the last few years, which allow the less-detailed, but much more rapid, characterization of single microbial cells. For example, Single Cell Raman Spectroscopy (SCRS) allows the direct measurement of intrinsic information about single cells in a non-invasive, label-free, and *in vivo* manner (Li et al., 2012; Smith et al., 2016). SCRS measures vibrations of biomolecules resulting from the inelastic scattering of incident laser light, producing a Raman spectrum, which is associated with a small physical volume (<1 μm^3^), of about the size of a bacterium. A typical single-cell Raman spectrum contains more than 1000 bands that can be assigned to different cellular compounds such as nucleic acids, protein, carbohydrates and lipids. With this information, SCRS enables the characterization of different cell types and can show physiological and phenotypic changes in living single cells. At its inception, the SCRS technique was afflicted by weak Raman signal and significant difficulty in the interpretation of the spectral data, however, recent work on multi-laser beams techniques like the Coherent anti-Stokes Raman Spectroscopy (CARS) (Min et al., 2011) and stimulated Raman spectroscopy (SRS) (Freudiger et al., 2011) has allowed a three order of magnitude increase in the strength of the signal, and significant improvements in signal interpretation. Any of these techniques could be routinely used to complement single-cell omics analysis. Prior to carrying out such an analysis on a particular microorganism in a soil thin section, one could ascertain whether its Raman spectrum is similar to one obtained for another organism already analyzed. In the affirmative, there may not be a need to perform a full single-cell omics protocol, resulting in considerable time saving.

## Conclusion

The key take-home message of this article is visualized in **Figure 5**. It presents our assessment of progress achieved to date toward what we view as the ultimate objective of the research about emerging soil microbial processes, namely the development of macroscopic measurement techniques that would provide us with the information needed to make reasonably accurate predictions. This **Figure 5** contains good news and bad news. The good news is that we have made significant progress. For forty years after prominent microbiologists argued in the mid 1960s that the *quantitative* microscale description of soil microbial processes was essential, the lack of suitable measurement techniques prevented the research from advancing at all. As a result, in spite of the publication of numerous articles on soil OM and on microbial processes in soils, very little progress has been achieved since the 1960s on several key questions in these areas. In the past 15 years, major technological breakthroughs have changed all that, with the result that our understanding of soils at the microscale has improved significantly on a number of fronts, experimentally as well as in terms of computer modeling. The bad news is that progress is very uneven. At the extremes of the spectrum, whereas research on the physical characteristics of soils at the microscale is moving full speed ahead, the (arguably more complicated) experimental observation of microbial processes is lagging far behind, casting doubt on the soundness on some of the extensive modeling that has been carried out in this field over the last decade, and hindering the needed integration of physical, (bio)chemical, and microbiological perspectives. Clearly, there is still a long way before reaching the holy grail, with many daunting challenges on the different paths leading to it.

There are reasons to be optimistic, however, and not to be intimidated by these challenges. For one thing, technological breakthroughs did not stop a decade ago. New measuring devices and new technologies in other respects as well (e.g., single-cell “omics”) are being developed and, for some of them, even getting commercialized, which should lead to many quantum leaps in our ability to carry out microscale measurements in soils. In addition, one can always hope that as we run more and more experiments to try to understand the emergent microbial properties of soils, someone will come up with an empirical equation that will provide a simple answer to all the questions we have at the moment, a little bit like what Henry Darcy did in his day for water movement in sand filters. Such an empirical description, if and when it becomes available, would completely change the game plan. But in the meantime, we need to keep in mind that we do not really have a choice but to move forward, no matter how challenging that might be. As was mentioned at the beginning of this article, the unresolved questions the research addresses are the object of extreme societal concern and it is not overblown to consider that they need to be answered urgently if we ultimately want humanity to survive. This message is not yet understood by decision makers in most countries, but as time goes by and it becomes more and more urgent to get answers, we should hope that even politicians will realize it will be in everyone’s best interest to devote to this research more than the shoe-string budgets that have been allocated to it so far.

## Data Availability

No new data were generated during the writing of this article.

## Author Contributions

PCB initiated the writing of this article, based on in-depth conversations he had over an extended period of time with all co-authors, and he revised several successive drafts of the text, after receiving substantial input from everyone (and very helpful suggestions from the associate editor and reviewers). All authors have approved publication of the final version.

## Conflict of Interest Statement

The authors declare that the research was conducted in the absence of any commercial or financial relationships that could be construed as a potential conflict of interest. The reviewer PN and handling Editor declared their shared affiliation.

## Footnotes

1 There is one exception to this general statement, in the case of some oxisols in tropical areas, such as the La Selva soil investigated by Radulovich et al. (1992). This soil, containing 95% kaolinite clay, is made of “pseudo-sand” microaggregates that are very regular in shape and size, so that it is possible in practice to repack the soil in a state that is very close to what it was originally.

2 K-edge is the binding energy of the K shell electron of an atom. There is a sudden increase in the attenuation coefficient of photons occurring at a photon energy just above the binding energy of the K shell electron of the atoms interacting with the photons.

3 http://www.palabos.org

4 http://www.openlb.net

5 Anyone who is planning to simulate soils by using glass beads should have a long look at some of the images in Foster (1988) and in later work that has followed on his footsteps!

6 As an example of this type of repeated headlines, Charles (2018), on the National Public Radio website in the United States, describes the “Earth Microbiome Project,” an initiative started a decade earlier, as a “new approach” to opening up the soil microbial blackbox.
