# Supplementary material for: Emergent Properties of Microbial Activity in Heterogeneous Soil Microenvironments: Different Research Approaches Are Slowly Converging, Yet Major Challenges Remain
Source: Front Microbiol. 2018 Aug 27;9:1929. doi: 10.3389/fmicb.2018.01929 (PMC6119716; doi:10.3389/fmicb.2018.01929)
Supplement: Supplementary file 1 [file Data_Sheet_1.pdf]

frontiers
in Microbiology

Supplemental Information

Emergent properties of microbial activity in heterogeneous soil microenvironments: Different research approaches are slowly converging, yet major challenges remain

Philippe C. Baveye[1]*, Wilfred Otten[2], Alexandra Kravchenko[3], María Balseiro Romero[1,4], Éléonore Beckers[5], Maha Chalhoub[6], Christophe Darnault[7], Thilo Eickhorst[8], Patricia Garnier[6], Simona Hapca[9], Olivier Monga[10], Carsten Müller[11], Naoise Nunan[12], Valérie Pot[6], Steffen Schlüter[13], Hannes Schmidt[14], Hans-Jörg Vogel[15]

[1] UMR ECOSYS, AgroParisTech, Université Paris-Saclay, Avenue Lucien Brétignières, Thiverval-Grignon 78850, France.

[2] School of Water, Energy and Environment, Cranfield University, Cranfield MK430AL, United Kingdom.

[3]Department of Plant, Soil and Microbial Science, Michigan State University, 1066 Bogue street, East Lansing, Michigan 48824, U.S.A.

[4]Department of Soil Science and Agricultural Chemistry, Centre for Research in Environmental Technologies (CRETUS), Universidade de Santiago de Compostela, 15782 Santiago de Compostela, Spain

[5] Soil–Water–Plant Exchanges, Terra Research Centre, BIOSE, Gembloux Agro-Bio Tech, University of Liège, Passage des Déportés 2, 5030 Gembloux, Belgium

[6] UMR ECOSYS, INRA, Université Paris-Saclay, Avenue Lucien Brétignières, Thiverval-Grignon 78850, France.

[7]Laboratory of Hydrogeoscience and Biological Engineering, L.G. Rich Environmental Laboratory, Department of Environmental Engineering and Earth Sciences, Clemson University, Anderson, SC 29625, United States

[8]Faculty 2 (Biology/Chemistry), University of Bremen, Bremen, Germany

[9] Dundee Epidemiology and Biostatistics Unit, School of Medicine, Dundee University, Kirsty Semple Way, Dundee DD24BF, United Kingdom.

[10]IRD, F-93143 Bondy Cedex, France.

[11]Lehrstuhl für Bodenkunde, Technical University of Munich, Emil-Ramann Strasse 2, 85354 Freising, Germany.

[12]Institute of Ecology and Environmental Sciences - Paris, Sorbonne Universités, UPMC Univ Paris 06-CNRS-IRD-INRA-P7-UPEC, 4 place Jussieu, 75005 Paris, France

[13]Soil System Sciences, Helmholtz-Zentrum für Umweltforschung GmbH - UFZ, Leipzig, Germany.

[14]Terrestrial Ecosystem Research, Department of Microbiology and Ecosystem Science, Research Network 'Chemistry meets Microbiology', University of Vienna, Althanstrasse 14, A-1090 Vienna, Austria

[15]Institute of Soil Science and Plant Nutrition, Martin Luther University of Halle-Wittenberg, Germany.

ADDITIONAL FIGURES

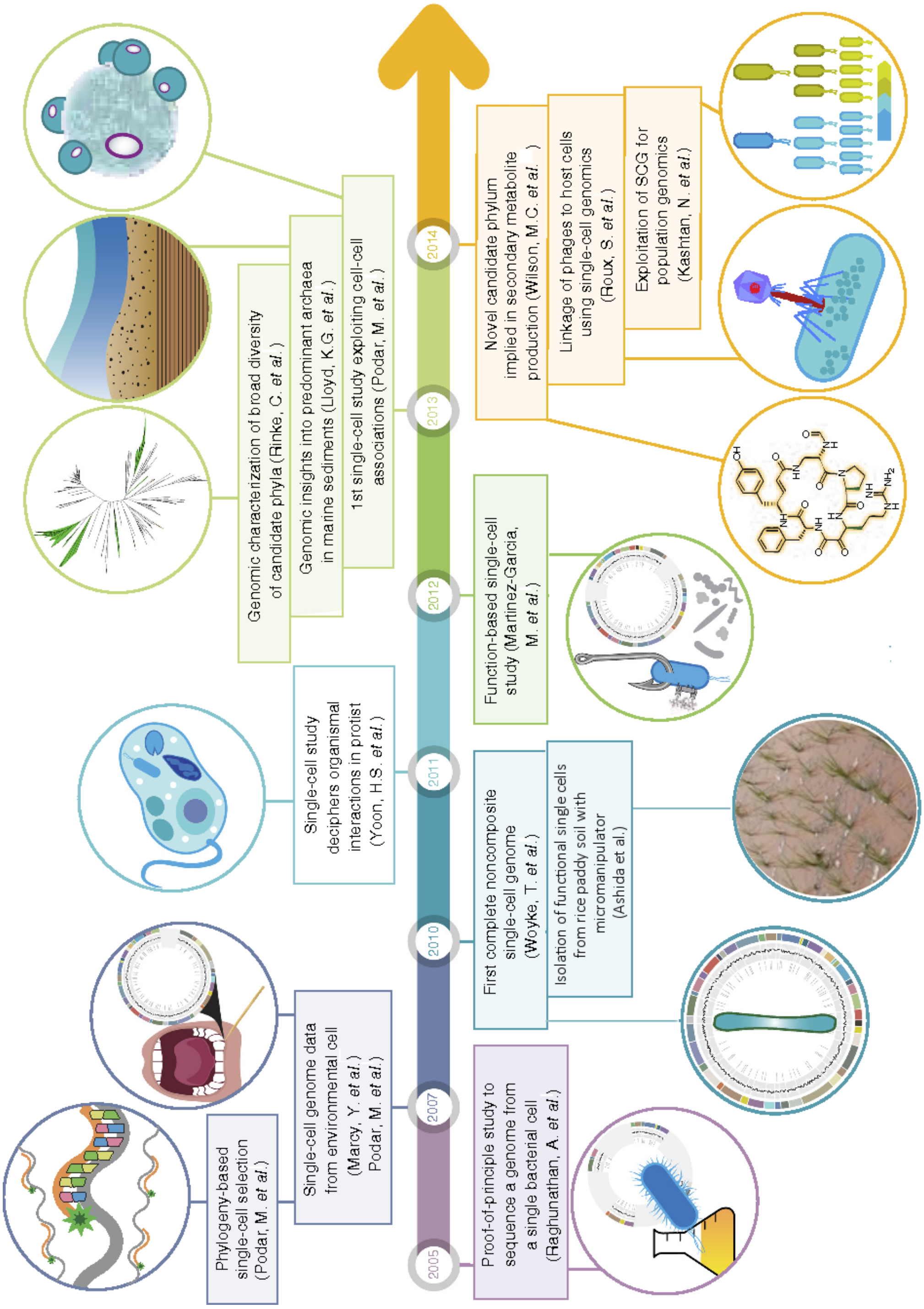

Figure S1. Timeline of early scientific milestones in single-cell microbial sequencing, modified from Woyke et al (2017). In addition to the original timeline of Woyke et al. (2017), Ashida et al.'s (2010) research has been added, related to the use of a micromanipulator to isolate individual cells from a sample of rice paddy soil.

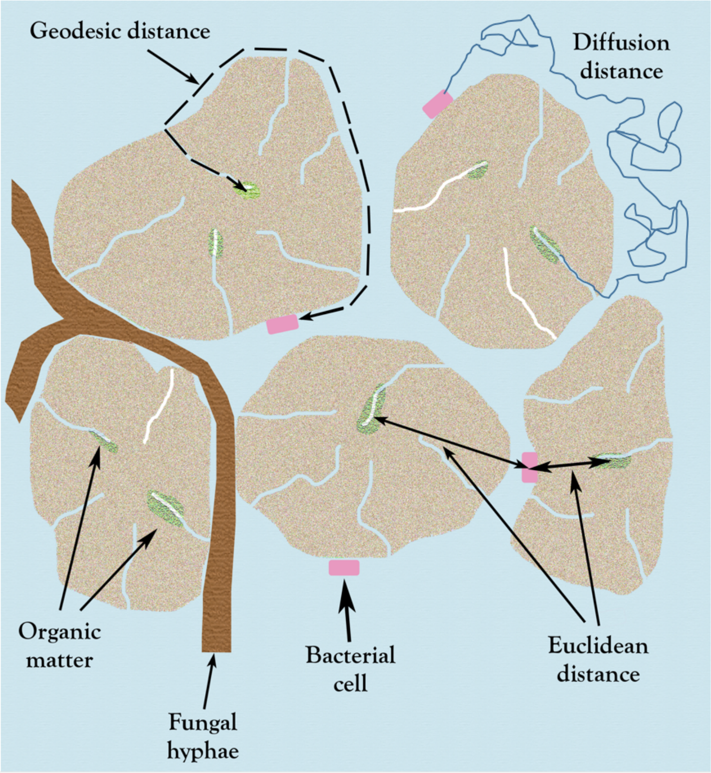

Figure S2. Schematic representation of the distribution of bacterial cells, fungal hyphae, and organic matter in the pore space of a soil, as well as three metrics used to account for the degree of physical separation between them. Euclidean distances can be easily calculated between bacteria or fungi and individual blobs of organic matter, even when there is no path through connected pore space between them. The "geodesic" distance, on the other hand, requires the existence of a connected path. Finally, the diffusion distance also requires the existence of a connected path, but takes into account the diffusion of dissolved organic matter, exoenzymes, or cells.

APPENDIX 1

Emergent properties

The term “emergence” has been the object of significant debate and controversy over the last few decades. “Despite its ubiquity and importance, emergence is an enigmatic and recondite topic, more to be wondered at than analyzed...”, wrote Holland (1990) not that many years ago. Indeed, the literature on emergent properties tends to be very confusing, with a single term being used sometimes to refer to different concepts, while different terms are used to describe the same notion.

Fortunately, since the early 90s, the philosophical reflection on emergence has been intense, and significant effort has gone into making the terminology more precise. An excellent summary of some of the key concepts associated with “emergence” is provided by Rastetter and Vallino (2015). These authors distinguish between “teleological” emergence, “chaotic” emergence, and “mechanistic” emergence. Under “teleological” emergence, objects at higher levels of organization acquire emergent properties that are not explainable in terms of the interactions among their constituent parts. There must therefore be some external causative agent acting toward the emergence of these

properties, or they arise spontaneously only in the context of the higher-level object. On the contrary, "chaotic" emergence does not at all ascribe causation to external causes, but assumes that emergent properties arise from nonlinear interactions within a system that are so sensitive to initial conditions to defy any kind of accurate prediction of system response. Likewise, "mechanistic" emergence refers to behaviors of a system that result from system-level interactions and feedbacks among the parts of the system, but it does not require sensitivity to initial conditions, and therefore it allows prediction of system responses based on information about what goes on inside the system. In that sense, mechanistic emergence includes chaotic emergence as a special case.

In addition to different definitions being used in connection with the term "emergence", two aphorisms seem to have become intimately associated with the concept of emergent properties over the years. The first is that "the whole is greater than the sum of its parts" (see, e.g., Addiscott, 2011), which is in many ways a direct rejection of reductionism "à la Descartes" (Dorit, 2011). The second aphorism is that "properties of the whole are unpredictable even from complete knowledge of its parts." Both of these statements, clearly, seem antithetical to a research program like the one described in this article, starting at the microscale to eventually understand better the macroscopic behavior of soils. Rastetter and Vallino (2015) view these aphorisms as "vestigial appendages of teleological emergence, which they accurately describe" and they consider that for the other concepts of emergence, these two aphorisms are misleading and foster confusion. In the case of mechanistic emergence, which is particularly relevant to macroscopic soil properties, these authors point out that, actually, "the whole is *less* than the sum of its parts." A prototypical example of this kind of simplification is Clapeyron's ideal gas law. Even though it is still, and probably always will be, impossible to describe exactly the behavior of a few hundred gas molecules, the deceptively simple ideal gas law allows this description for systems containing extremely large numbers of gas molecules. In partially-saturated soils, the conceptually simple Richardson-Richards equation (Raats and Knight, 2018) is also very successful at describing fluid flow at the macroscopic scale, in spite of the extreme complexity of the pore space at the microscopic scale (e.g., Kasteel et al., 2000). Taking nutrient cycling in soils as another example, Rastetter and Vallino (2015) argue that the cycles for different nutrients are not independent from one another; under normal conditions, it would be impossible to accumulate nitrogen within soils without also having an adequate stock of phosphorous and other essential elements. In other words, because of interactions and feedbacks among microscale processes in soils, there are bounds on the range of combinations that materialize. What thus emerges is a reality that is *simpler* than what it could have been, given the many constituents that soils comprise.

Emergence or self-organization?

In this context of macroscopic behaviors of soils being simpler than what they could theoretically have been, various authors have preferred to invoke the notion of "self-organization" rather than that of emergence (Smagin, 1989; Hallet, 1990; Phillips, 1995, 2000; Manson, 2001; Young and Crawford, 2004; Lavelle et al., 2007; Barot et al., 2007; Ebrahimi and Or, 2016; Lavelle et al., 2016; Tecon and Or, 2017a,b). Camazine et al. (2003) define self-organization as "a process in which pattern at the global level of a system emerges solely from numerous interactions among the lower-level components of the system." With different words but the same general idea, Di

Marzo Serugendo et al. (2005) define self-organization as "the mechanism or the process enabling a system to change its organization without explicit external command during its execution time".

Use of this concept in relation to soils allows publications to be connected to a huge body of physics literature on self-organization, which some might see as a definite benefit. However, even though Di Marzo Serugendo et al. (2005) argue convincingly that for a variety of systems, "the appearance of emergence implies that the system also exhibits self-organization", a view adhered to by Addiscott (2011) as well in the case of soils, it is not clear that both concepts apply equally well to soils, which are by their very nature open systems, subjected constantly to various *external* stimuli and environmental constraints. It seems reasonable, under these conditions, to consider that the absence of "explicit external command" is more than doubtful in the case of soils and that the relevance of the notion of *self*-organization becomes somewhat fuzzy. In addition, whenever the concept of self-organization has been applied to soils, it has often been associated with specific organisms, typically bacteria (e.g., Ebrahimi and Or, 2016), neglecting the fact that they not only compete with other organisms (fungi, notably) for nutrients, but also are preyed upon by various predators, e.g., protozoans, and are parasited by viruses. Therefore, it seems inappropriate to refer to soil bacteria, or any other individual group of organisms for that matter, as *self*-organized... However, bearing in mind that bacterial communities in soils are probably quite small and numerous, it is possible to hold another viewpoint on this issue of self-organization. One might presume that a sizeable portion of bacterial communities in soils are free from competition with fungi and are protected from predation by the limiting size of the surrounding porosity, leaving the possibility that, at least in terms of interactions with other organisms, they be truly *self*-organized. Be that as it may, the fact that soils are open systems subjected to many external stimuli, still remains an obstacle for the notion of self-organization, and we shall therefore stick to using the term "emergence" in the following, emphasizing the idea of simplification that it conveys.

REFERENCES

Addiscott, T. M. (2011). Emergence or self-organization? Look to the soil population. *Communicative*

Barot, S., Rossi, J. P., & Lavelle, P. (2007). Self-organization in a simple consumer-resource system, the example of earthworms. *Soil Biology & Biochemistry, 39*(9), 2230-2240. doi:10.1016/j.soilbio.2007.03.021

Camazine, S., Deneubourg, J.-L., Franks, N. R., Sneyd, J., Theraulaz, G., & Bonabeau, E. (2003). *Self-organization in biological systems*. Princeton, New Jersey: Princeton University Press.

Di Marzo Serugendo, G., Gleizes, M.-P., & Karageorgos, A. (2005). Self-organization in multi-agent systems. *The Knowledge Engineering Review, 20*(2), 165-189.

Dorit, R. L. (2011). The Humpty-Dumpty problem. *American Scientist, 99*(4), 293-295.

Ebrahimi, A., & Or, D. (2016). Microbial community dynamics in soil aggregates shape biogeochemical gas fluxes from soil profiles - upscaling an aggregate biophysical model. *Global Change Biology, 22*(9), 3141-3156. doi:10.1111/gcb.13345

Hallet, B. (1990). Self-organization in freezing soils: From microscopic ice lenses to patterned ground. *Canadian Journal of Physics, 68*(9), 842-852.

Kasteel, R., Vogel, H. J., & Roth, K. (2000). From local hydraulic properties to effective transport in soil. *European Journal of Soil Science, 51*(1), 81-91. doi:10.1046/j.1365-2389.2000.00282.x

Lavelle, P., Barot, S., Blouin, M., Decaens, T., Jimenez, J. J., & Jouquet, P. (2007). Earthworms as key actors in self-organized soil systems. In K. Cuddington, J. E. Byers, W. G. Wilson, & A. Hastings (Eds.), *Ecosystem Engineers: Plants to Protists* (pp. 77-106).

Lavelle, P., Spain, A., Blouin, M., Brown, G., Decaens, T., Grimaldi, M., . . . Zangerle, A. (2016). Ecosystem engineers in a self-organized soil: A review of concepts and future research Questions. *Soil Science, 181*(3-4), 91-109. doi:10.1097/ss.0000000000000155

Manson, S. M. (2001). Simplifying complexity: a review of complexity theory. *Geoforum, 32*(3), 405-414. doi:10.1016/s0016-7185(00)00035-x

Phillips, J. D. (1995). Self-organization and landscape evolution. *Progress in Physical Geography, 19*(3), 309-321. doi:10.1177/030913339501900301

Phillips, J. D. (2000). Signatures of divergence and self-organization in soils and weathering profiles. *Journal of Geology, 108*(1), 91-102. doi:10.1086/314386

Raats, P. A. C., & Knight, J. H. (2018). The contributions of Lewis Fry Richardson to drainage theory, soil physics, and the soil-plant-atmosphere continuum. *Frontiers in Environmental Science, 6*(13). doi:10.3389/fenvs.2018.00013

Rastetter, E. B., & Vallino, J. J. (2015). Ecosystem's 80th and the Reemergence of Emergence. *Ecosystems, 18*(5), 735-739. doi:10.1007/s10021-015-9893-6

Smagin, A. V. (1989). Soil as a product of biogeocenosis self-organization. *Doklady Akademii Nauk Sssr, 308*(3), 729-731.

Tecon, R., & Or, D. (2017). Cooperation in carbon source degradation shapes spatial self-organization of microbial consortia on hydrated surfaces. *Scientific Reports, 7*. doi:10.1038/srep43726

Tecon, R., & Or, D. (2017). Biophysical processes supporting the diversity of microbial life in soil. *Fems Microbiology Reviews, 41*(5), 599-623. doi:10.1093/femsre/fux039

Young, I. M., & Crawford, J. W. (2004). Interactions and self-organization in the soil-microbe complex. *Science, 304*(5677), 1634-1637. doi:10.1126/science.1097394
